# Laser-Induced Creation of Coherent V2 Centers in Bulk-Grown Silicon Carbide

**DOI:** 10.1021/acs.nanolett.6c01807

**Published:** 2026-07-09

**Authors:** Laurens J. Feije, Gerben M. Timmer, Yihong Hu, Rana Karababa, Guido L. van de Stolpe, Tobias Martens, Sjoerd J. H. Loenen, Theo B. A. Durant, Antariksha Das, Aaron M. Day, Evelyn L. Hu, Tim H. Taminiau

**Affiliations:** † QuTech, Delft University of Technology, PO Box 5046, 2600 GA Delft, The Netherlands; ‡ Kavli Institute of Nanoscience Delft, Delft University of Technology, PO Box 5046, 2600 GA Delft, The Netherlands; § John A. Paulson School of Engineering and Applied Sciences, 1812 Harvard University, Cambridge, Massachusetts 02138, United States

**Keywords:** Color centers, Laser-induced defect creation, Nanophotonic structures, Silicon carbide, Quantum networks, Solid-state qubits

## Abstract

Solid-state spin defects are promising qubits for quantum network nodes. A key challenge toward larger networks is creating defects with high yield into nanophotonic devices while maintaining good optical and spin properties. Here, we demonstrate the creation of single V2 centers in nanopillars fabricated from commercial bulk-grown 4H-silicon carbide using a pulsed above-bandgap (UV) laser. We observe an 11-fold increase in the V2 center occurrence after UV laser illumination. These laser-induced V2 centers exhibit narrow optical line widths and spectral diffusion rates comparable to naturally occurring V2 centers in nanopillars of the same material. Furthermore, we measure a spin coherence time of $T_{2}^{DD}=3.6\left(3\right)\;\mathrm{ms}$ under dynamical decoupling, consistent with dephasing by the nuclear-spin bath. This demonstration of the in situ, postfabrication generation of coherent V2 centers in nanostructures in widely available bulk-grown 4H-SiC shows the potential for above-bandgap laser illumination for scalable defect creation in integrated photonic devices.

## Graphical abstract

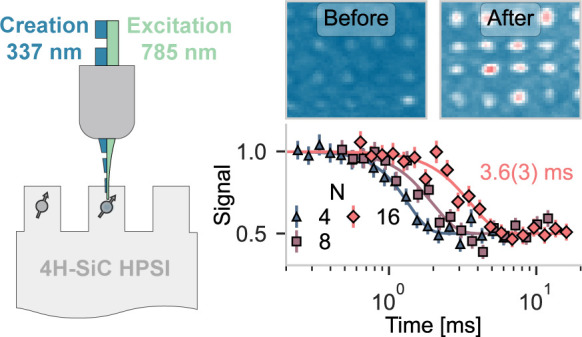

Solid-state spin defects provide promising qubits for quantum networks, as they combine high-fidelity spin-photon entanglement with access to nuclear spins with long coherence times.^1–3^ Their compatibility with integrated photonics offers a scalable path toward large-scale quantum networks.^4–6^ A central challenge is the deterministic placement of defects within subwavelength nanophotonic structures while preserving good optical and spin coherence.^7–10^

Silicon carbide (4H-SiC) hosts a variety of optically active spin defects, including silicon vacancies, divacancies, the vanadium defect and the NV center.^10–20^ The negatively charged single k-site silicon vacancy center (V2) is a promising candidate as long spin coherence times and near-lifetime-limited optical transitions, required for remote entanglement generation, have been demonstrated in nanophotonic devices.^21–23^

Several methods have successfully demonstrated the creation of localized single V2 centers, such as masked He^+^ ion implantation, which has been shown to yield V2 centers with good optical and spin coherence,^21,24^ and focused ion beam (FIB) implantation,^25–27^ including with in situ monitoring.^28^ Pulsed-laser writing is a compelling alternative that provides defect creation with good spatial precision, typically on the order of 100–200 nm, after device fabrication.^29–32^ In addition, laser writing is compatible with in situ monitoring,^29^ potentially enabling deterministic defect creation. Pioneering experiments in 4H-SiC have shown that below-bandgap laser writing can create color centers in bulk,^31–33^ while above-bandgap laser writing has further enabled the creation of V2 centers in 1D nanophotonic crystal cavities.^30^

However, the optical and spin coherence of single laser-written defects in 4H-SiC, which are essential for many quantum applications, have thus far remained unexplored.

Here, we demonstrate laser-induced creation of V2 centers in nanopillars with preserved optical and spin coherence. We use an above-bandgap laser and commercial bulk-grown high-purity semi-insulating (HPSI) 4H-SiC, widely available as 4–6 in. wafers.

We first identify the pulse energy window that enables defect formation without inducing surface amorphization. Subsequently, we show the creation of single laser-induced V2 centers and characterize their optical properties. We show near-lifetime-limited optical line widths and slow spectral diffusion rates, similar to naturally occurring V2 centers in the same type of nanopillars. Finally, we demonstrate long electron-spin coherence times (up to 3.6(3) ms) for laser-induced V2 centers, similar to values reported in high-quality epitaxial 4H-SiC.^11,34^ The demonstration of spatially selective defect creation after device fabrication, with good optical and spin coherence in nanostructures made of commercial bulk-grown wafers, indicates that above-bandgap pulsed-laser illumination is a promising route toward scalable generation of coherent V2 centers in 4H-SiC devices.

We use an above-bandgap (337 nm, 3.68 eV) pulsed (3 ns) laser (similar as Day et al.^30^) focused on the sample via a 0.7 NA objective and investigate single-pulse defect creation in c-plane HPSI 4H-SiC with a bandgap of 3.26 eV^35^ (Figure 1a,b, see Supporting Note S1 for sample preparation and Supporting Note S16 for the optical setups). Given the high efficiency of electron–hole pair generation at above-bandgap excitation, the material is susceptible to irreversible damage under intense irradiation.^30^ To mitigate this, we first determine the laser-induced amorphization threshold (LIAT)^36,37^ for both nanopillars and bulk HPSI 4H-SiC. This is achieved by exposing one hundred discrete spots per pulse energy (see Supporting Note S2). After UV illumination, scanning electron microscopy (SEM) is used to quantify the number of sites exhibiting resolvable surface amorphization (Supporting Note S3). We note that our analysis is limited to SEM-detectable surface amorphization, which excludes subsurface lattice damage.

**Figure 1:**
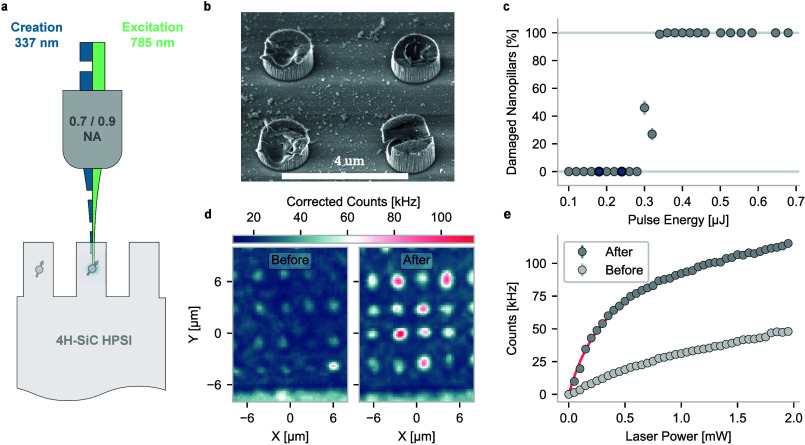
**Laser-induced defect creation using an above-bandgap pulsed laser. a)** Schematic of the laser-induced creation and detection of defects in HPSI 4H-SiC. A 0.7 NA objective focuses an above-bandgap (337 nm) pulsed (3 ns) laser onto the center of the nanopillar to create defects. For imaging, we use a 0.9 NA objective and a 785 nm laser for off-resonant excitation. **b)** Scanning electron microscopy image of the HPSI amorphized nanopillars that were exposed to a single UV pulse with energies higher than the LIAT (0.28 μJ). The nanopillars are ∼1.2 μm in diameter and ∼4 μm spaced from center to center. **c)** Amorphization statistics of nanopillars using a single pulse. For each pulse energy, one hundred nanopillars were each illuminated with a single pulse, and the percentage of nanopillars that showed visible damage is indicated on the *y*-axis. Blue dots indicate 0.18 μJ and 0.24 μJ, which are used for Figure 2 and Figure 3. See Figure S1 for SEM pictures of amorphization. **d)** 2D PL scans before and after a single UV pulse of 0.24 μJ at each pillar. The counts for both measurements were corrected to have the same background counts in bulk close to the nanopillars (before counts ×1.524, and after counts ×0.656, see Supporting Note S5 for correction method). **e)** Fluorescence saturation measurements before and after a single UV pulse of 0.24 μJ on the same pillar.

Despite being limited to surface-level observations, our analysis enables the identification of three distinct regimes: no visible amorphization, probabilistic amorphization, and deterministic amorphization, observed in both the nanopillars (Figure 1c) and bulk material (Supporting Note S3). We find that the LIAT for nanopillars (>0.28μJ) is lower compared to bulk (>0.32μJ) , showing that it is dependent on the nanophotonic structure (consistent with previous studies^38^).

With the LIAT determined, we verify that defect creation is possible at energies below this threshold. We perform off-resonant (785 nm) 2D photoluminescence (PL) scans before and after a single UV laser pulse for pulse energies below the LIAT, employing a 0.9 NA objective to improve collection efficiency (Supporting Note S4). An example of one of these 2D PL scans can be found in Figure 1d. For pulse energies ≥0.18 μJ, the 2D PL scans show an increase in PL for nearly all nanopillars (see Supporting Note S5), indicating subsurface lattice deformation or laser-induced defect generation.^39^ Next, we perform fluorescence saturation measurements under off-resonant laser excitation on each individual nanopillar. These measurements reveal early saturation effects, associated with quantum-defect emission, for UV pulse energies ≥0.18 μJ, see Supporting Note S6. This suggests that laser-induced defect creation occurs over a broad parameter space, providing a usable energy window (0.18 μJ to 0.28 μJ) in which color centers can be introduced without inducing amorphization.

To further characterize UV-pulse-induced defect creation, we perform fluorescence saturation measurements on an additional 64 nanopillars before and after a single 0.18 μJ UV pulse (low-probability regime), and on 64 nanopillars exposed to a 0.24 μJ pulse (higher-probability regime). An example of a nanopillar fluorescence saturation measurement before and after a single pulse can be seen in Figure 1e.

To determine whether the observed increase in PL arises from the presence of V2 centers, we search for the characteristic zero-phonon line emission, corresponding to the A1 and A2 optical transitions, through photoluminescence excitation spectroscopy (PLE) spectra at 4 K.^17,40^ We then compare the occurrence of V2 center peaks after exposure with a single UV pulse (0.18 μJ or 0.24 μJ) relative to the natural occurrence (without UV pulse). We fit all PLE spectra (see Supporting Note S7, Supporting Note S8 and Supporting Note S9) and compare the frequencies and amplitudes of the fitted peaks (example of PLE in Figure 2a). Evaluating the percentage of V2 centers with peak amplitudes above a certain threshold reveals a significantly larger number of high-count-rate V2 centers in UV-exposed nanopillars (see Figure 2b). Relative to the brightest V2 center in the nonexposed nanopillars (0.76(2) kHz), nanopillars exposed to a 0.18 μJ or 0.24 μJ UV pulse contain roughly 3 and 11 times more V2 centers that reach or exceed this brightness, respectively. Furthermore, the brightest V2 center found in the UV-exposed nanopillars has a count rate of 2.11(4) kHz, approximately a factor of 2.8 higher than the brightest emitter in the nonexposed nanopillars.

**Figure 2:**
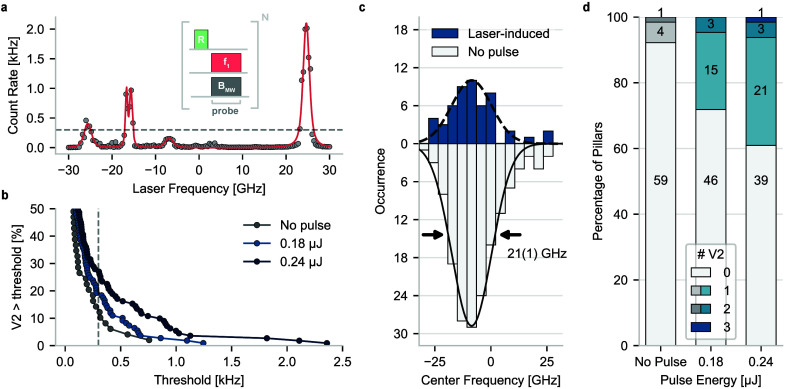
**Photoluminescence excitation spectroscopy (PLE) and number of V2 centers. a)** Single PLE of a nanopillar with three V2 centers passing a threshold (dashed gray line) at 0.3 kHz. Laser frequency is offset from 327.112 THz. Inset shows the experimental sequence, green indicates an off-resonant (repump) pulse (10 μs, 10 μW), and red indicates a single resonant laser (2 ms, 100 nW). Gray indicates microwaves (MW) at (70 MHz) to counteract spin pumping. **b)** Percentage of V2 centers whose fitted peak amplitude exceeds the count-rate threshold, shown separately for nanopillars exposed to a UV pulse (0.18 μJ and 0.24 μJ) and those left unexposed; in each condition, *n* = 64 nanopillars are examined. We find a maximum count rate for the nonexposed V2 center of 0.76 kHz. For this threshold, we also observe 3 and 11 times more V2 centers with the same or higher count rate at UV laser powers of 0.18 μJ and 0.24 μJ, respectively. **c**) Ensemble inhomogeneous distribution of all V2 centers in unexposed nanopillars (bottom gray bars). A Gaussian fit yields an fwhm of the inhomogeneous distribution of 22(1) GHz. The blue bars indicate the frequencies of the V2 centers in the UV-exposed nanopillars that pass the threshold of 0.3 kHz (this threshold sets a cutoff to suppress bulk-V2 contributions, also indicated by the dotted line in b)). The black dashed line shows the (scaled) Gaussian fit of the unexposed inhomogeneous distribution as a guide to the eye. **d)** Number of nanopillars that contain either 0, 1, 2, or 3 V2 centers that pass the 0.3 kHz threshold. Nanopillars exposed to the UV laser are shown in shades of blue, while natural (unexposed) V2 centers are shown in shades of gray. The numbers inside the bars show how many pillars contain that specific number of V2 centers.

Because weak background emission can originate from defects outside the nanopillars, we restrict our analysis to V2 centers with a PLE amplitude above 0.3 kHz (dotted line, Figure 2a,b). This threshold ensures that only bright, well-collected V2 centers (most likely located near the nanopillar center, see Supporting Note S4) are included.

Using the 0.3 kHz threshold, we find that the frequencies of the V2 centers in the UV-exposed nanopillars follow the same ensemble inhomogeneous distribution as the natural V2 center population (Figure 2c). This suggests that the UV pulse does not introduce significant strain or amorphization in the vicinity of the laser-induced V2 centers.^22,41,42^ Furthermore, we quantify the probability of inducing only one bright V2 center for the two UV pulse energies (Figure 2d). After a single 0.18 μJ pulse, ∼23% of the nanopillars contain one bright V2 center, increasing to ∼33% for 0.24 μJ. For comparison, this is observed in only ∼6% of nonexposed nanopillars. These results show that the UV-pulse strongly increases the number of V2 centers, even though the exact origin of each V2 center, whether created by the UV-pulse or naturally present, cannot be determined unambiguously. As we will show below, the optical properties of V2 centers with and without UV illumination are very similar, so this ambiguity does not affect the conclusion that the UV pulse induces high-quality V2 centers. In the remainder of this work, we will therefore refer to V2 centers in UV-exposed nanopillars as laser-induced V2 centers and in nonexposed nanopillars as natural V2 centers.

Next, we characterize the optical properties of the laser-induced V2 centers and compare them to natural V2 centers using the check-probe spectroscopy methodology of van de Stolpe et al.^23^ We characterized 24 individual V2 centers, including 3 natural (circles in Figure 3a,b) and 21 laser-induced centers. Among the laser-induced centers, 10 were exposed to a single UV pulse with an energy of 0.18 μJ (squares), while 11 were exposed to a single pulse with an energy of 0.24 μJ (triangles).

**Figure 3:**
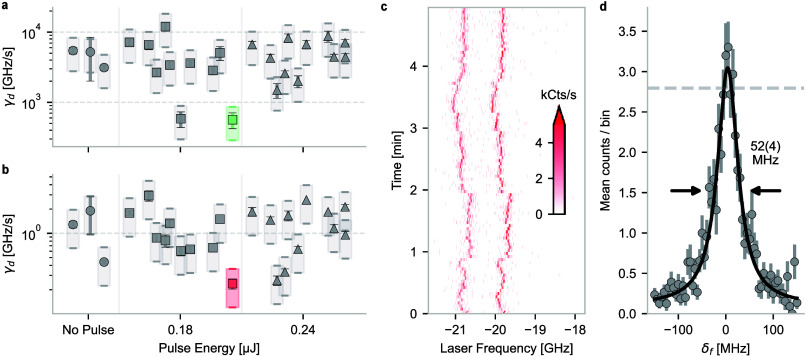
**V2 optical properties and spectral diffusion. a)** Fitted spectral diffusion constant under off-resonant excitation for natural V2 centers (circles) and laser-induced V2 centers (squares and triangles, 0.18 μJ and 0.24 μJ respectively). We extract γ_d_ using the methodology described in van de Stolpe et al.,^23^ assuming a homogeneous line width (fwhm) of 36 MHz. Because the fitted γ_d_ depends on this homogeneous line width (see Supporting Note S10), and this line width was not measured for each individual V2 center, we indicate the spread in γ_d_ for a plausible range of line widths (1 ≤ Γ/Γ_lifetime_ ≤ 3, see Supporting Note S11). γ_d_ of the red square indicates the same V2 center as the green square in b) and data in c) and d). **b)** Fitted spectral diffusion constants under resonant excitation. **c)** Scanning laser PLE over 5 min of the V2 center highlighted in a) and b) (Pillar 14, Figure S7). The laser is on for a total of 334 ms per scan with a power at the objective of 20 nW. **d)** Check-probe PLE (see van de Stolpe et al.^23^ and Supporting Note S11 for methodology) with fwhm = 52(4)­MHz indicating similar optical line width (Γ/Γ_lifetime_ = 2.21(4), see Figure S17) as natural V2 centers in this material (see Figure S16). Check-probe PLE was performed at a magnetic field of ∼20 G aligned along the crystal *c*-axis (see Supporting Note S12).

The measured spectral diffusion rates under resonant and off-resonant excitation are shown in Figure 3a,b. This reveals a 3–4 orders-of-magnitude difference in spectral diffusion between resonant and off-resonant laser light, consistent with previous work.^23^ Additionally, we observe a significant spread in spectral diffusion rates, indicating that each V2 center experiences a distinct local charge environment. Importantly, we find no significant difference in the spectral diffusion rates between laser-induced and natural V2 centers; the two groups exhibit similar overall distributions. This suggests that the single UV pulse does not substantially alter the effective local charge environment, and that the impurities of the HPSI material or the embedding in a nanopillar, are still the main cause of spectral diffusion.^42^ Within the distribution of spectral diffusion constants (γ_d_) for laser-induced V2 centers, some V2 centers remain particularly stable, exhibiting low γ_d_ even under off-resonant excitation (green and red markers in Figure 3a,b).

We perform two additional types of photoluminescence excitation (PLE) measurements. First, we observe the spectral wandering (Figure 3c) by scanning a resonant laser continuously while applying microwaves; second, we use a check-probe type PLE on the A1 transition to approximate the homogeneous line width (Figure 3d).^23^ By sweeping the threshold for the check-probe PLE, we can minimize the residual broadening due to imperfect initialization and compare this homogeneous line width to the lifetime-limited line width (see Supporting Note S11). We find line widths satisfying 1 ≤ Γ/Γ_lifetime_ ≤ 4 for all measured V2 centers, indicating that there is still some broadening (either residual (in)­homogeneous broadening, or power broadening). The line widths for the natural V2 centers are similar to the laser-induced V2 centers, indicating that the UV pulse does not significantly lower the optical coherence of the V2 centers and, importantly, are compatible with remote entanglement generation under modest time filtering.^43^

In addition to optical coherence, the electron spin coherence is a crucial component for utilizing laser-induced V2 centers in sensing, quantum information processing, and quantum network applications. We investigate the spin properties of two single laser-induced V2 centers, which exhibits relatively low spectral diffusion (γ_d_) under both resonant and off-resonant excitation (pillar 9 and 14; see Figure S7 for location details, Supporting Note S13 for optical properties of V2 center in pillar 9, Figure 3c,d for optical properties of V2 center in pillar 14 and Figure S23 for spin properties of V2 center in pillar 14). Note that this optical selection might already introduce a bias in the electron spin environment and thus, potentially in the electron spin coherence.

First, we operate at a magnetic field of ∼40 G. This ensures that the Kramers degeneracy within the $\pm \frac{1}{2}$ and $\pm \frac{3}{2}$ spin eigenstates is lifted, and that the ground or excited state level crossings are sufficiently detuned.^42,44,45^ Figure 4a shows Rabi oscillations for driving the ground state $\left|m_{s}=+\frac{1}{2}\right\rangle ↔\left|m_{s}=+\frac{3}{2}\right\rangle$ transition of the V2 center found in pillar 9. On the same V2 center, we perform electron spin resonance (ESR), see Figure 4b (details of the sequence can be found in Supporting Note S14). ESR reveals a strongly coupled nuclear spin, with a hyperfine coupling of 2.19(5) MHz. This coupling is consistent with either a coupled ^13^C or ^29^Si nuclear spin as previously observed and reported,^46^ and accounts for the detuning visible in the Rabi chevron pattern (Figure 4a and Supporting Note S14). We perform Ramsey measurements to extract the spin-dephasing time $T_{2}^{*}$ , finding $T_{2}^{*}=0.9\left(1\right)\;\mu s$ (Figure 4c).

**Figure 4:**
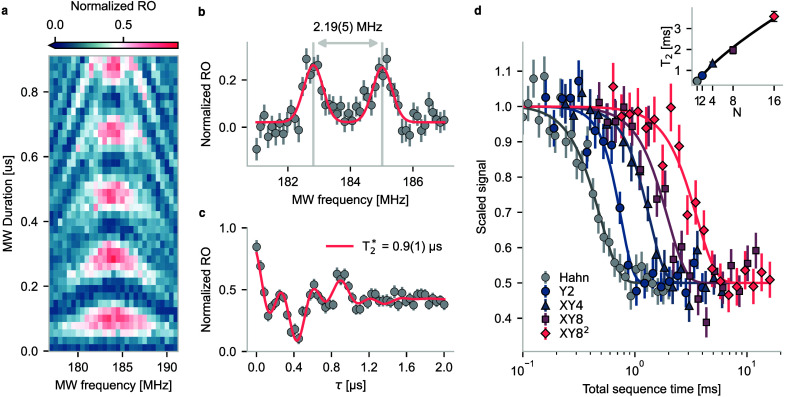
**Electron spin properties. a)** Rabi chevron pattern from the V2 center in pillar 9. Details on the measurement sequence and normalization procedure are elaborated in Supporting Note S14. **b)** Electron-spin-resonance of the same V2 center revealing a strongly coupled nuclear spin with hyperfine splitting of 2.19(5) MHz (red line). **c)** Detuned Ramsey measurement on the same V2 center with *f*_MW_ = 181.8 MHz. Red line indicates a fit to an oscillation with two frequency components and a Gaussian decay, which translates to a hyperfine splitting of *f*_HF_ = 2.08(7)­MHz, consistent with the measurement in b), and $T_{2}^{*}=0.9\left(1\right)\;\mu s$ . **d)** Scaled Hahn-echo (*N* = 1) and dynamical decoupling (*N* = 2 to *N* = 16) measurements on the V2 center in pillar 14 (Figure S7) with no (resolvable) strongly coupled nuclear spin (see Figure S23 for Chevron, ESR and Ramsey) at a magnetic field of ∼1300 G. We find a $T_{2}^{Hahn}=0.49\left(2\right)\;\mathrm{ms}$ and extend the coherence time to $T_{2}^{DD}=3.6\left(3\right)\;\mathrm{ms}$ with two XY8 sequences. The inset shows *T*_2_ as a function of the number of π-pulses *N*, fitted to a power function *T*_2_ = β ·*N* ^α^ (excluding Hahn-echo) from which we extract α = 0.73(4) and β = 0.46(4) ms.

Next, we operate at a higher magnetic field (∼1300 G) to suppress decoherence from anisotropic hyperfine interactions with the spin bath. We perform Hahn-echo and dynamical decoupling (DD) measurements on the V2 center in pillar 14, which has no strongly coupled nuclear spins. We measure a Hahn-echo coherence time of $T_{2}^{Hahn}=0.49\left(2\right)\;\mathrm{ms}$ and extend the coherence up to $T_{2}^{DD}=3.6\left(3\right)\;\mathrm{ms}$ using two consecutive XY8 sequences (Figure 4d).

The $T_{2}^{DD}$ values display a power-law dependence on the number of decoupling pulses, with a fitted exponent of α = 0.73(4). This is slightly higher than the α = 2/3 scaling associated with a Lorentzian noise spectrum $\left(∼\frac{1}{\omega^{2}}\right)$ , characteristic of Ornstein–Uhlenbeck dynamics,^47^ indicating a slight deviation from a single-Lorentzian noise spectrum toward lower frequency noise.^48^ Despite the relatively high nitrogen concentration of (∼1.1 × 10^15^ cm^–3^, see Supporting Note S1) our $T_{2}^{*}$ and $T_{2}^{Hahn}$ are comparable to values reported for natural-isotope-abundance epitaxially grown 4H-SiC with doping levels 2 orders of magnitude lower.^11,34,49–52^ This suggests that neither the doping level nor the laser-writing method limits the spin coherence. These results show that commercially available wafers combined with laser-induced defect creation might provide a viable path toward quantum technologies based on color centers in 4H-SiC.

In this work, we studied the creation of laser-induced V2 centers in 4H-SiC nanopillars in bulk-grown SiC. We observe narrow optical line widths that are compatible with remote entanglement generation,^43^ and long electron-spin coherence times consistent with dephasing by the nuclear spin bath,^11^ although potential contributions from electron spin impurities cannot be excluded.

Looking forward, while here we focus on the formation of V2 centers in bulk-grown 4H-SiC wafers, the methods employed here can potentially be extended to high-quality, isotopically engineered, epitaxially grown 4H-SiC,^53–57^ and as well to other defect centers, such as V1 centers (h-site $V_{\mathrm{Si}}^{-}$ ),^40^ divacancies,^14^ and non-native atom-related complexes like NV centers.^15^ A systematic exploration of defect formation, density, and properties using above-bandgap laser writing, across different irradiation conditions and writing parameters, can enable deeper insight into the underlying material science and the mechanisms of defect creation, including potential local annealing or activation effects,^58^ in different types of SiC and nanostructures.

Additionally, monitoring the photoluminescence between laser pulses might enable the deterministic creation of a desired number of defects.^29^ Large-scale fabrication methods, such as Silicon-Carbide-on-Insulator^5^ or angled etching,^10^ would open the door to complex integrated nanophotonic structures, including photonic crystal cavities. Together with the laser-induced creation of high-quality V2 centers in commercial bulk-grown SiC wafers presented here, this combination might enable a path toward efficient, high-quality defect creation in scalable quantum devices.

## Supplementary Material



## Data Availability

All data underlying the study are available on the open 4TU data server: 10.4121/be9e8523-5fb9-4861-abad-95b71231c086. Code used to operate the experiments is available on request.

## Notes

Sample preparation; pulsed UV experiments; examples of SEM images used to determine the laser-induced amorphization threshold; simulated comparison of collection efficiency with different NA objectives; additional data on room-temperature photoluminescence and laser saturation measurements; PLE V2 selection; locations of the observed V2 centers together with the photoluminescence excitation measurements; Hanbury Brown Twiss experiments for several V2 centers; methods for characterizing the spectral diffusion rate and homogeneous line widths, together with data of several V2 centers; magnetic field alignment; pulse scheme and simulations of the spin measurements; spin coherence at low magnetic field; schematics of optical setups (PDF)
