# Supplementary material for: Laser-Induced Creation of Coherent V2 Centers in Bulk-Grown Silicon Carbide
Source: Nano Lett. 2026 Jul 9;26(28):9143–50. doi: 10.1021/acs.nanolett.6c01807 (PMC13397884; doi:10.1021/acs.nanolett.6c01807)
Supplement: Supplementary file 1 [file nl6c01807_si_001.pdf]

Laser-induced creation of coherent V2 centers in bulk-grown silicon carbide

L.J. Feije,[1,2,*] G.M. Timmer,[1,2,*] Y. Hu,[1,2] R. Karababa,[1,2] G.L. van de Stolpe,[1,2] T. Martens,[1,2]
S.J.H. Loenen,[1,2] T.B.A. Durant,[1,2] A. Das,[1,2] A.M. Day,[3] E.L. Hu,[3] and T.H. Taminiau[1,2,†]

[1] *QuTech, Delft University of Technology, PO Box 5046, 2600 GA Delft, The Netherlands*
[2] *Kavli Institute of Nanoscience Delft, Delft University of Technology, PO Box 5046, 2600 GA Delft, The Netherlands*
[3] *John A. Paulson School of Engineering and Applied Sciences, Harvard University, Cambridge, MA, USA*

SUPPLEMENTARY INFORMATION

S1. Sample preparation

The sample was diced directly from a 6-inch High-Purity Semi-Insulating (HPSI) wafer obtained from Wolfspeed (model type W4TRG0R-N-0200). We note that the HPSI terminology originates from the silicon carbide electronics industry. In the quantum technology context considered here, this material has a significant amount of residual impurities (order ~$10^{15}\,\mathrm{cm^{-3}}$ according to Son et al.[1]) and is hence considered low material quality with respect to a concentration of ~$10^{13}\,\mathrm{cm^{-3}}$ typical for epitaxially grown layers on the c-axis of silicon carbide.[2,3] On a different sample, diced from a wafer of the same model type, secondary-ion mass spectrometry (SIMS) determined the nitrogen donor concentration as $[\mathrm{N}] = 1.1 \times 10^{15}\,\mathrm{cm^{-3}}$. In addition to intrinsic silicon vacancies, additional silicon vacancies were generated in this sample through a previous (unrelated) 2 MeV electron irradiation with a fluence of $2\times10^{13}\,\mathrm{cm^{-2}}$. The sample was annealed at 600 °C for 30 min in an Argon atmosphere. To enhance the optical collection efficiency and mitigate the unfavourable V2 dipole orientation for confocal access along the SiC growth axis (c-axis), we utilize nanopillars. Nanopillar fabrication begins with sputtering an aluminium oxide (Al_2O_3) mask on the SiC substrate, followed by spinning and patterning of AR-P 6200 e-beam resist (Allresist GmbH). The resist pattern is transferred into the Al_2O_3 using inductively coupled plasma reactive ion etching (ICP-RIE) with a BCl_3/Cl_2 plasma. Afterwards, the pattern is etched into the SiC using ICP-RIE and an SF_6/O_2 plasma. After ICP-RIE, the remaining Al_2O_3 was removed with hydrofluoric acid.

S2. Pulsed UV experiment

All pulsed UV experiments are performed at room temperature and ambient pressure. To determine the LIAT for bulk material, we define a grid of 100 spots (10 by 10) for each pulse energy. To avoid spatial and temporal biasing (e.g., alignment or temperature drifts), the pulse energies are varied in a randomized order and calibrated before each measurement. All spots are positioned within a region where alignment artifacts are negligible. The z-position of the objective is fine-tuned by scanning through different heights while applying a pulse energy slightly above the LIAT. We then use the z position that produces the largest amorphization on the surface (see S3).

The same procedure is used to determine the LIAT for nanopillars, with the grid consisting of 4 by 25 individual structures. The measurement process is fully automated, involving piezo movement of the objective and a single UV pulse. Due to grid interpolation and piezo positioner hysteresis, the UV pulse may not always focus in the exact center of each nanopillar. While this misalignment has minimal effect on the LIAT determination, it can slightly influence the resulting concentration of V2 centers.

S3. Laser-induced amorphisation threshold

Due to the efficient generation of electron-hole pairs using an above-bandgap laser, linear photo-ionization can generate a high density of free electrons. This can lead to the formation of a highly ionised plasma, potentially resulting in material damage[4–6]. Given the fixed pulse duration of 3 ns, we systematically vary the pulse energy of

* These authors contributed equally

† t.h.taminiau@tudelft.nl

single-shot exposures to determine the damage threshold. This threshold defines the upper bound of pulse energy used in subsequent investigations, referred to as the laser-induced amorphisation threshold (LIAT). The procedure is applied to both bulk 4H-SiC and nanostructured pillars (diameter ~1.2 µm, height ~1 µm) and followed by SEM inspection, see Fig. S1a-f.

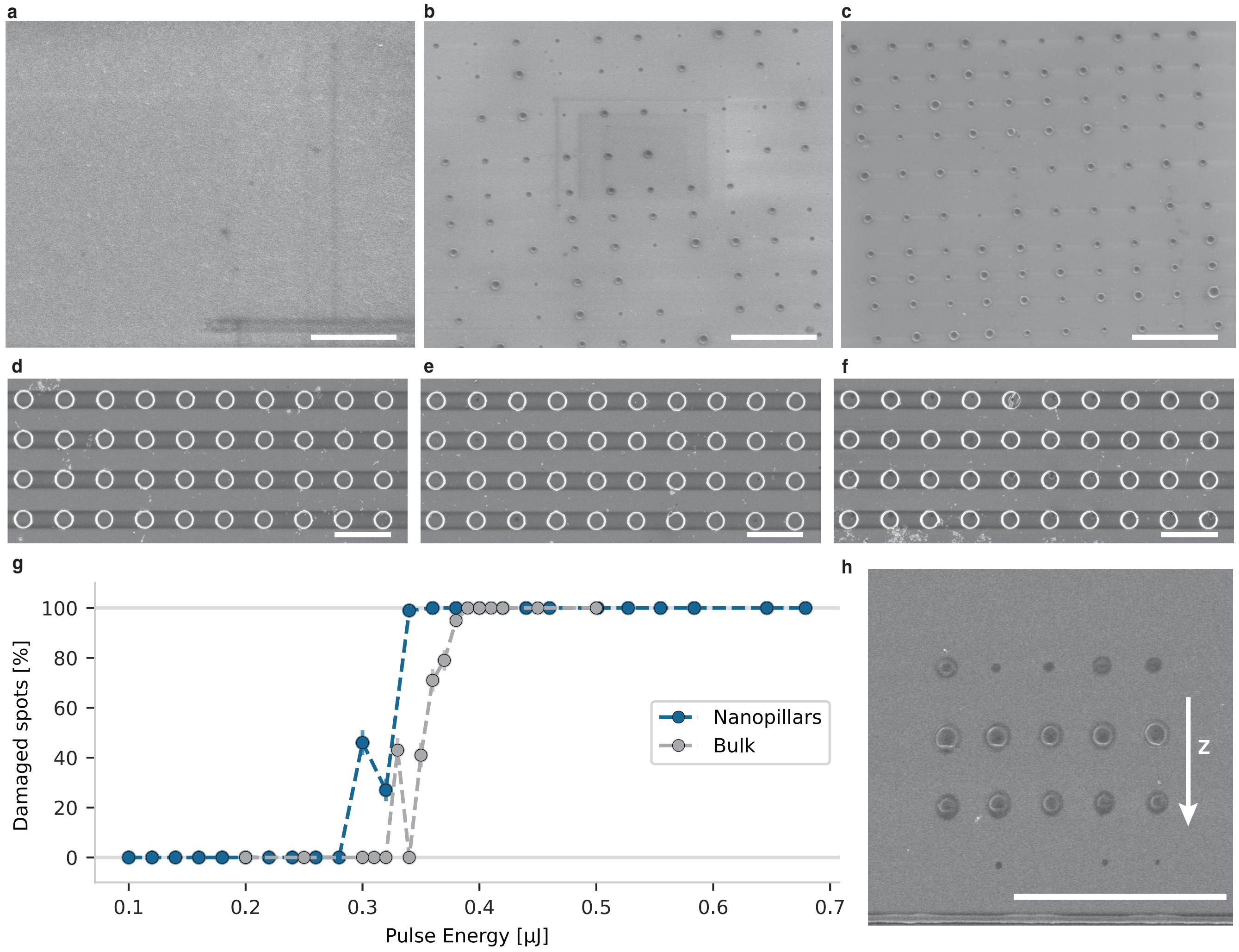

Fig. S1. **SEM of LIAT for bulk and nanopillars.** **a)** Bulk experiment with single pulse energy of 0.20 µJ, of a grid of 100 spots with no visible amorphisation. **b)** Bulk experiment with single pulse energy of 0.38 µJ with probabilistic (80%) amorphisation. **c)** Bulk experiment with single pulse energy of 0.40 µJ with 100% amorphisation. **d)** Nanopillar experiment with single pulse energy of 0.24 µJ of a grid of 100 spots with no visible amorphisation. **e)** Nanopillar experiment with single pulse energy of 0.3 µJ with probabilistic (46%) amorphisation. **f)** Nanopillar experiment with single pulse energy of 0.34 µJ with 100% amorphisation. **g)** The number of damaged sites for each pulse energy, for both bulk and nanopillars. **h)** Example of the focusing procedure of the UV laser. For each row, the z-position is slightly adjusted to compare the surface-damage diameter. In all the SEM images, the white bar indicates 5 µm.

Following SEM analysis, the number of damaged sites was quantified and plotted as a function of pulse energy (Fig. S1g), revealing the energy dependence of laser-induced damage thresholds across different surface morphologies. A distinctly non-linear response is observed in both the bulk material and the nanopillars. As we do not focus on the probabilistic armophization regime, we do not investigate this further.

Prior to each pulse-energy configuration, the UV laser focus was optimised on the substrate surface by adjusting the objective's z-axis, while using high-energy UV pulses of 0.45 µJ, to maintain consistent exposure parameters. As can be seen in Fig. S1h, the second row exhibits the largest surface-damage diameters, which we attribute to optimal focusing of the UV laser at the substrate surface during the experiment.

S4. Simulated collection efficiency

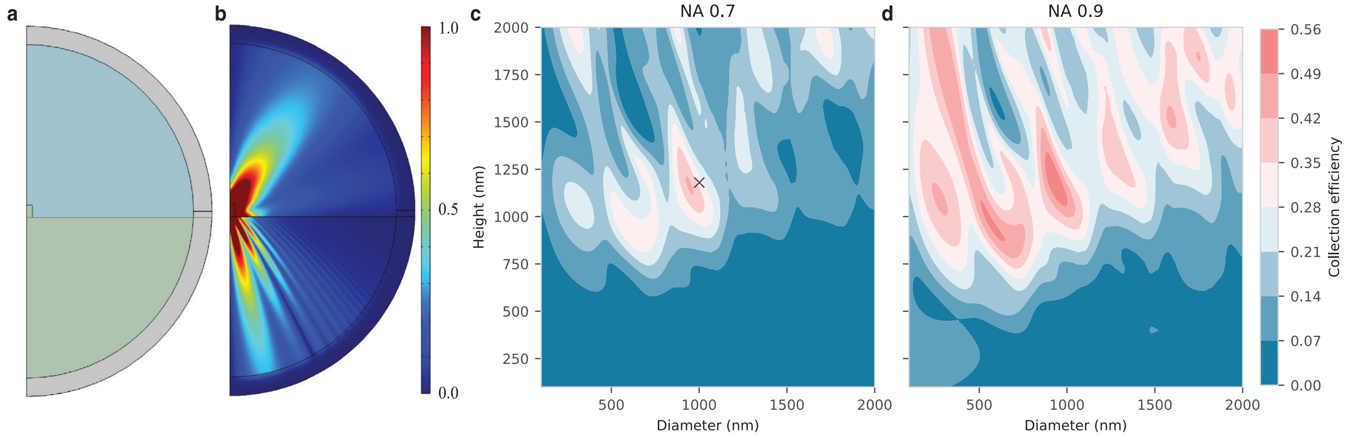

Fig. S2. **Simulated collection efficiency for nanopillars. a)** Geometry used to simulate the V2 center ZPL emission pattern in nanopillars using COMSOL Multiphysics 5.6. 2D axis-symmetry is applied to reduce computational time. The green area is specified as 4H-SiC ([7]: Î±-SiC; n(o) 0.488-1.064 µm), the blue area is specified as air, and the outer grey area is defined as a perfectly matched layer. In the center of the nanopillar, we place an artificial copper antenna to emulate the V2 center ZPL emission. **b)** The emission pattern of the V2 center in a nanopillar with dimensions as indicated with the black cross in **c**. For visualization purposes, the colour scale has been saturated to enhance the contrast and reveal the underlying emission pattern. **c),d)** The calculated collection efficiency for various nanopillar diameters and heights with a 0.7 and 0.9 NA objective, respectively.

The perpendicular optical dipole orientation of the V2 defect with respect to the surface (in c-plane 4H-SiC) limits the collection efficiency through an objective positioned above the sample. Nanopillars are an effective way to alleviate this limitation. To simulate the effects of a nanopillar structure, we use the 2D-axisymmetric electromagnetic waves, frequency-domain physics interface of COMSOL Multiphysics 5.6. Fig. S2a illustrates the geometry used. The green region is specified as 4H-SiC (Singh et al. 1971: Î±-SiC; n(o) 0.488-1.064 µm), the blue region is air, and the outer grey area is a perfectly matched layer. In the center of the nanopillar, an artificial copper antenna models the optical emission of a V2 defect, with a single frequency emission corresponding to the ZPL transition. The emission of a V2 defect in a nanopillar (dimensions indicated with the black cross in Fig. S2c) are shown in Fig. S2b. The collection efficiencies in Fig. S2c,d are calculated with:

$$\eta = \frac{P_{\text{objective}}}{P_{\text{tot}}}, \tag{S1}$$

with P_{tot} being the total power emitted by the artificial antenna and P_{obj} the power directed towards the objective, calculated as:

$$P_{\text{obj}} = \int_0^{\sin^{-1}(\text{NA})} P_{\text{boundary}}(\theta)\text{d}\theta, \tag{S2}$$

where P_{boundary} is the power normal to the boundary surface and NA = 0.7 and NA = 0.9 in Fig. S2c,d, respectively.

These simulations are performed only at the ZPL wavelength and with the emitter positioned at the center of the nanopillar. While a real V2 defect emits over a broader spectrum and may reside at different locations within the pillar, exploring these additional configurations lies beyond the scope of this work. The present model is therefore intended as a representative estimate to guide the nanopillar geometry rather than an exhaustive optimization.

S5. 2D-PL Before and after UV pulse

We perform 2D photoluminescence (PL) experiments on all nanopillars, followed by single UV pulse exposure, with varying pulse energy. Subsequently, we perform 2D PL scans again over the same nanopillars.
The 2D-PL measurements are performed with a different objective than the one used for the UV pulse, and exchanging

objectives leads to a small shift in collection efficiency. Therefore, in order to have a fair comparison between the before- and after-scans, we use the mean count rate of a reference bulk region (grey dashed line in S3) to establish a common baseline and extract the appropriate scaling factor between the two measurements. We use $\alpha = \sqrt{\mu_{\text{before}} \times \mu_{\text{after}}}$ (with μ the mean along the dashed grey line) and define the scaling factor for before and after as $\beta_{\text{before,after}} = \frac{\mu_{\text{before,after}}}{\alpha}$. We multiply all the counts in the before- and after-scans by β_{before} and β_{after}, respectively. Note that more nanopillars

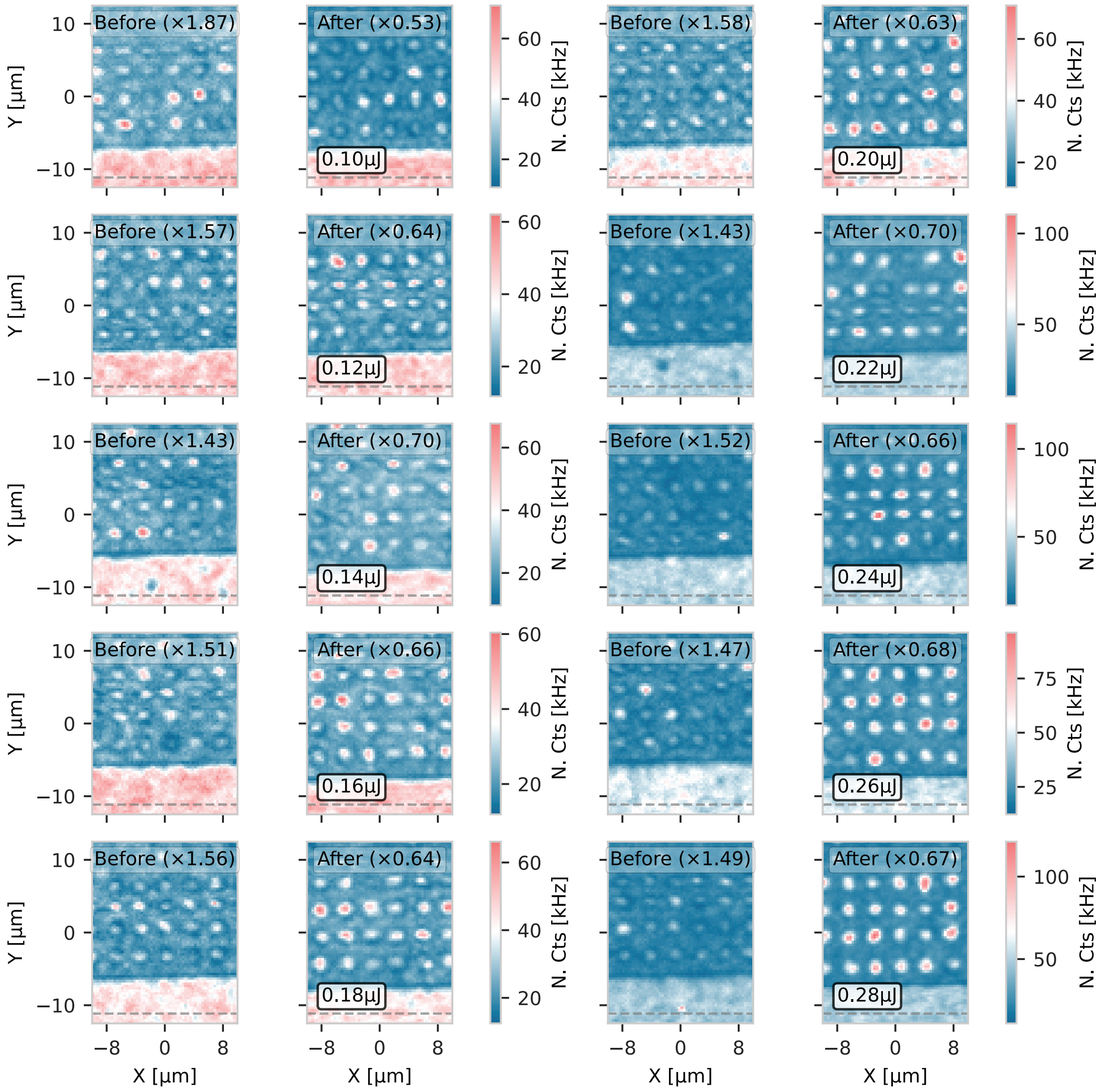

Fig. S3. **Normalised 2D PL before and after a single UV pulse for different pulse energies**. Here, the factor β is indicated in each 2D PL scan. The dashed grey line indicates the Y location, which is used to normalise the before- and after-PL counts with respect to each other.

were exposed to a UV pulse than the ~24 visible in each 2D-PL scan, and not all scans are included in this supplement. The scans shown are taken at approximately the same X–Y positions, although the exact center positions before and after exposure do not perfectly coincide.

At pulse energies of 0.16 µJ and above, we observe a clear increase in PL across multiple nanopillars, marking the onset of light-emitting defect formation. As the LIAT is approached, all nanopillars exhibit a substantial PL enhancement, suggesting the formation of additional emitters or non-visible (with SEM inspection) amorphization.

S6. Room-temperature laser saturation

At room temperature, we performed PL measurements on all UV-exposed nanopillars (100 nanopillars per pulse energy). For each nanopillar, we first optimize the objective position by applying 500 µW of off-resonant laser power and scan all 3 axis of the objective. Afterwards, we extract the PL counts at the optimal position and plot the distribution of maximum PL counts versus pulse energy in a violin plot Fig. S4. We observe that the level of PL counts and spread increases for pulse energies $\geq 0.18\,\mu J$. Next, we sweep the off-resonant laser power from 0 mW to

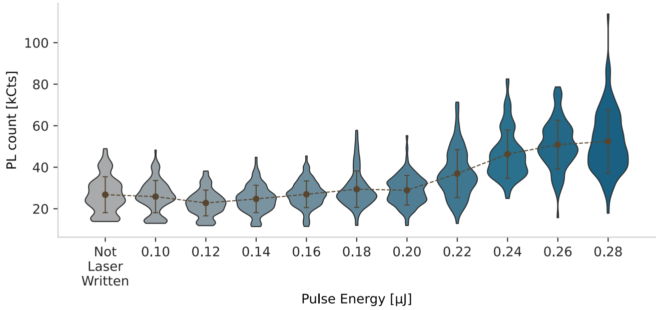

Fig. S4. **Distribution Photoluminescence:** Maximum PL signal after scanning X, Y and Z axis of the objective with an off-resonant laser power of 500 µJ. The violin plot displays the distribution (symmetric histogram) for each UV pulse energy.

2 mW and collect the PL (example in main text Fig. 1f). We fit the data to a saturation curve (PL from defect) + a linear term (background PL):

$$PL = B \cdot p + A \cdot \frac{p}{p + p_{\mathrm{sat}}}. \tag{S3}$$

Here, p indicates the off-resonant laser power and B, A and p_{sat} are fitting parameters. We then define ρ at p_{sat} as $\frac{A_{\mathrm{sat}}}{A_{\mathrm{sat}}+B_{\mathrm{sat}}}$, with $A_{\mathrm{sat}} = \frac{A}{2}$ and $B_{\mathrm{sat}} = B \cdot p_{\mathrm{sat}}$ which gives us the ratio between the signal and the background PL. We plot the distribution of ρ at p_{sat} for each UV pulse energy in the violin plots displayed in Fig. S5. We observe a decrease in the lower tail of the distribution ($\rho \leq 0.6$) for an increase in UV pulse energy. Furthermore, we notice a slight increase of ρ at p_{sat} and a small decrease in variance for pulse energies $\geq 0.16\,\mu J$. However, there is also a slight decrease in ρ for pulse energies $\geq 0.24\,\mu J$, hinting at an increase in background fluorescence.

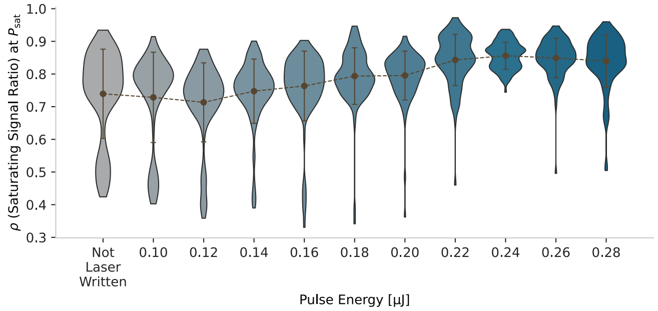

Fig. S5. **Signal vs. background (ρ) at saturation power.** Violin plot distribution of the fraction between the amplitude of the saturating signal vs the background signal at fitted saturation power.

S7. Automated PLE V2 selection

We measure PLE spectra on 192 nanopillars and fit them automatically. We apply microwaves to the V2 centers using a bondwire (~20 µm to 50 µm away from the laser-induced V2 centers) to efficiently counter spin-pumping.

For the non-UV-exposed and UV-exposed nanopillars, we perform the following measurement sequence.

1. Optimize the x,y,z position of the focal point to maximize the amount of counts under off-resonant excitation for each nanopillar.

2. Perform photoluminescence excitation (PLE) measurement by scanning a resonant laser over 60 GHz (while continuously applying microwaves at 70 MHz) centered around the observed inhomogeneous distribution of V2 centers.

3. Fit a Voigt profile to all observed peaks in the PLE spectra and store the center frequency and amplitude of the fit. To avoid double counting (A1 and A2 transition of a single V2 center) and fitting errors, we set the additional constrains to the fit: distance between fitted peaks >2 GHz and the FWHM of the fit should be larger than 50 MHz and lower than 3000 MHz and have a minimal count rate of 0.03 kHz.

S8. Location of V2 centers

To count the number of V2 centers, we perform PLE measurements on each nanopillar according to the scheme indicated in the inset of Fig. 2a. The method is explained in the methods section of the main text. In Fig. S6, Fig. S7, Fig. S8, we show the spatial location and fitted PLE data for the unexposed, 0.18 µJ, and 0.24 µJ nanopillars, respectively, which hosts 'bright V2 centers' passing the threshold. There seems to be no direct relationship between the spatial location of the nanopillars and the number of 'bright V2 centers'. All nanopillars are within ~50 µm of the bondwire, and for each group of 64 nanopillars, the optical path was optimized on collection efficiency. Furthermore, the nanopillars shown in this section are all located within an area of 250 µm×20 µm.

All PLE spectra for nanopillars that contain V2 centers and did not pass the threshold can be found in Fig. S9, Fig. S10, Fig. S11

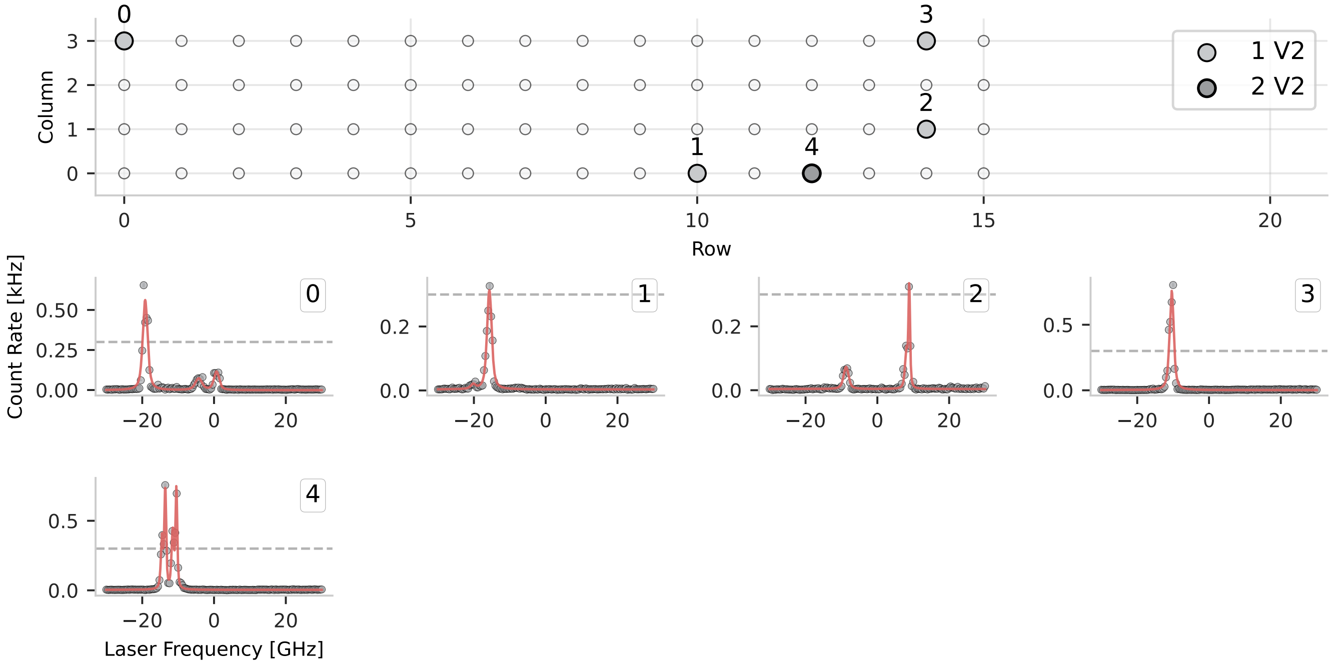

Fig. S6. **Photoluminescence excitation of 64 unexposed nanopillars.** The white circles indicate a raster of nanopillars. The grey circles indicate nanopillars with V2 centers passing the 0.3 kHz threshold (grey dashed line), and their respective PLEs are shown below. Note that for all the PLE figures the relative laser frequency was scanned from -30 GHz to 30 GHz (offset is 327.112 THz)

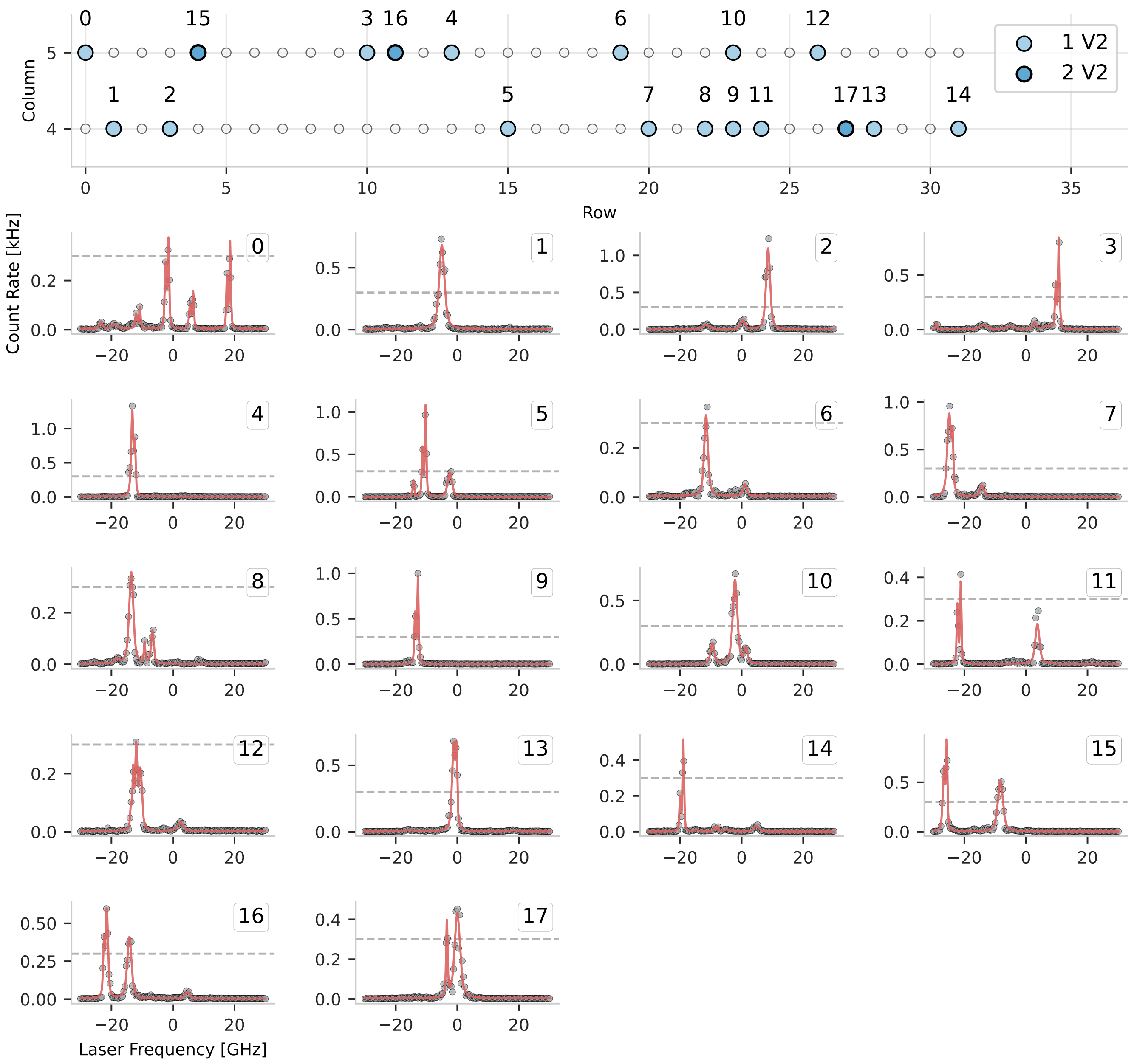

Fig. S7. **Photoluminescence excitation of 64 nanopillars exposed to a single pulse of** 0.18 µJ. The white circles indicate a raster of nanopillars. The blue circles indicate nanopillars with V2 centers passing the 0.3 kHz threshold (grey dashed line), and their respective PLEs are shown below. Note that for all the PLE figures the relative laser frequency was scanned from -30 GHz to 30 GHz (offset is 327.112 THz)

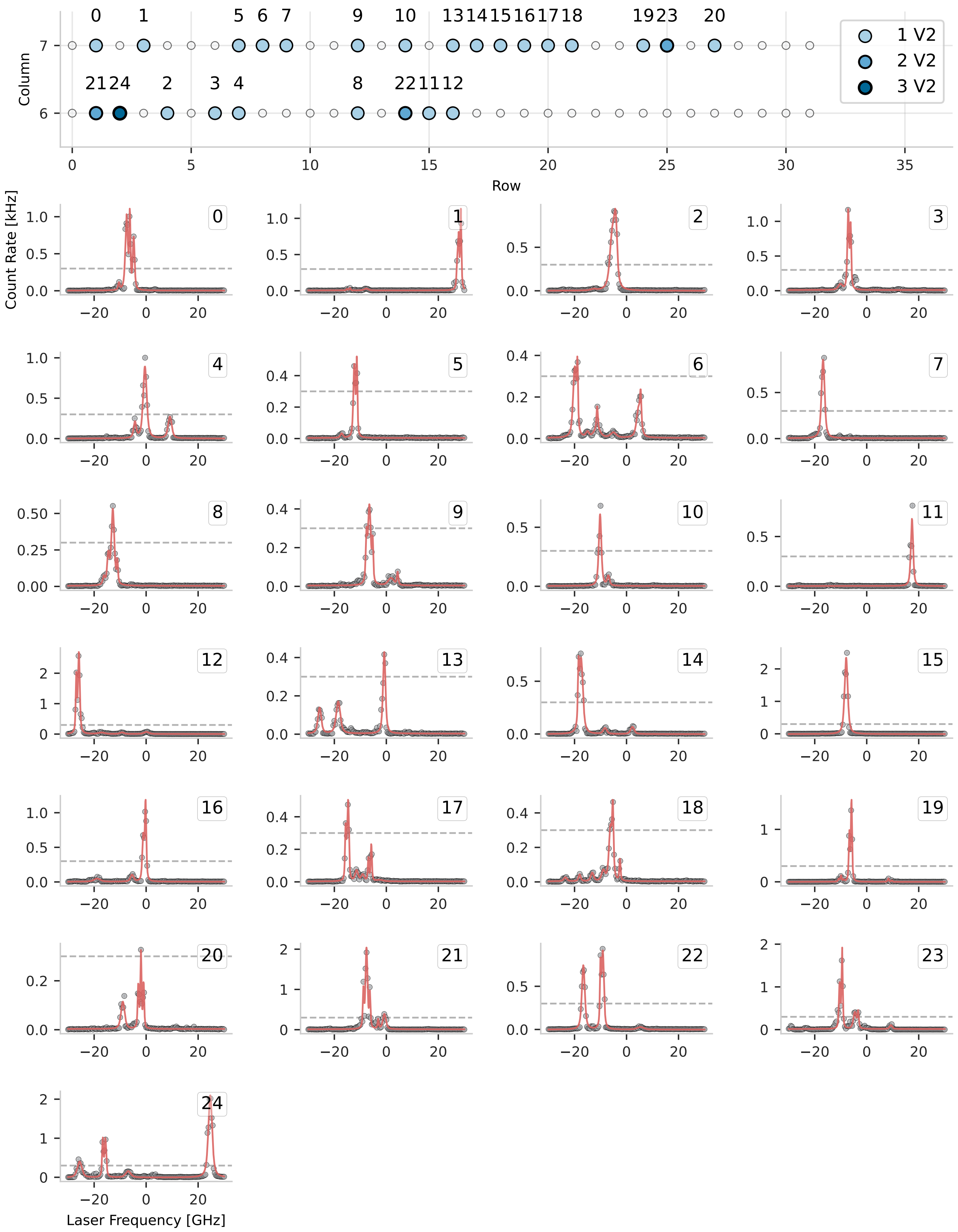

Fig. S8. **Photoluminescence excitation of 64 nanopillars exposed to a single pulse of** $0.24\,\mu J$**.** The white circles indicate a raster of nanopillars. The blue circles indicate nanopillars with V2 centers passing the 0.3 kHz threshold (grey dashed line), and their respective PLEs are shown below. Note that for all the PLE figures the relative laser frequency was scanned from -30 GHz to 30 GHz (offset is 327.112 THz)

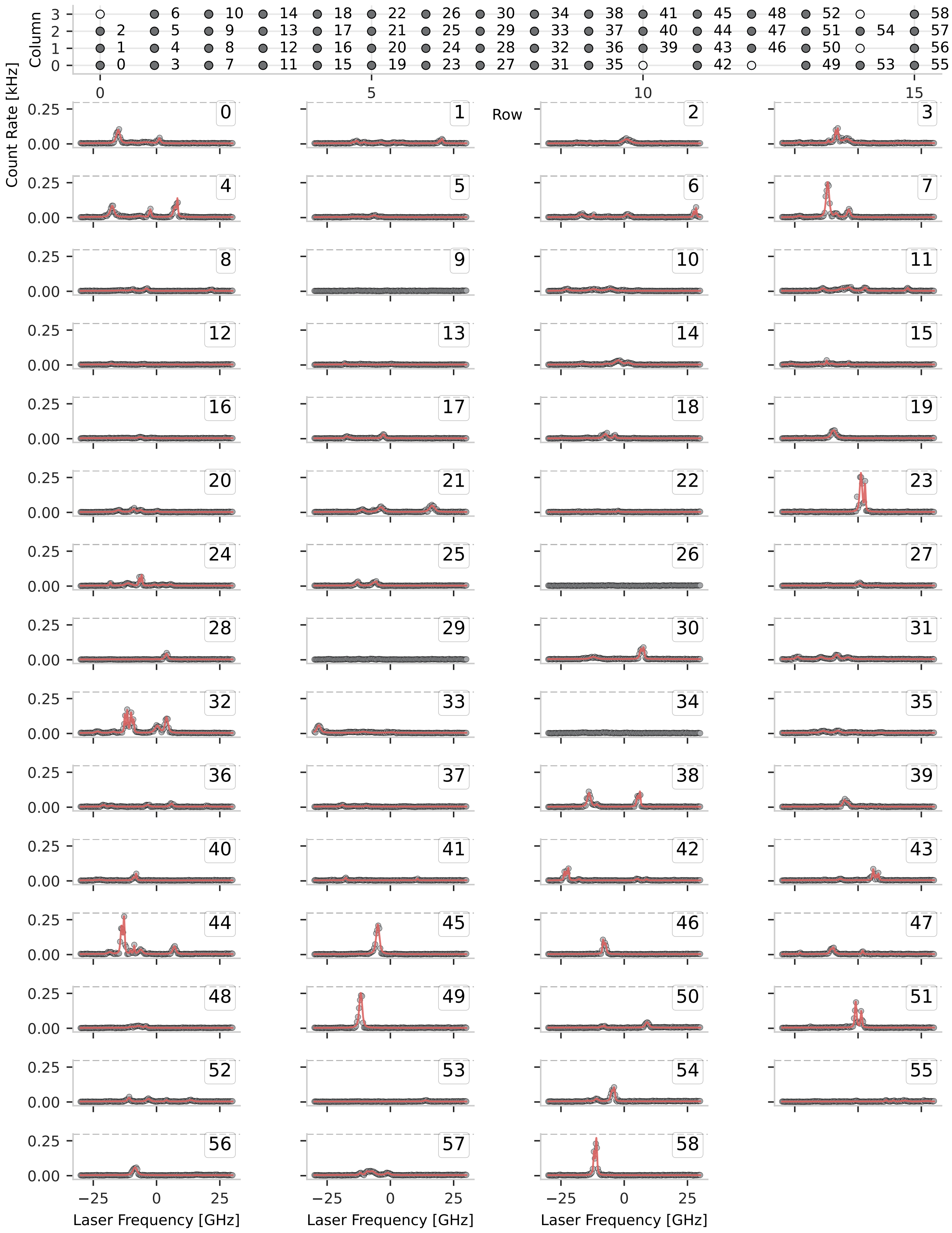

Fig. S9. **Photoluminescence excitation of 64 unexposed nanopillars.** The grey circles indicate nanopillars with V2 centers not passing the 0.3 kHz threshold (grey dashed line), and their respective PLEs are shown below. Note that for all the PLE figures the relative laser frequency was scanned from -30 GHz to 30 GHz (offset is 327.112 THz)

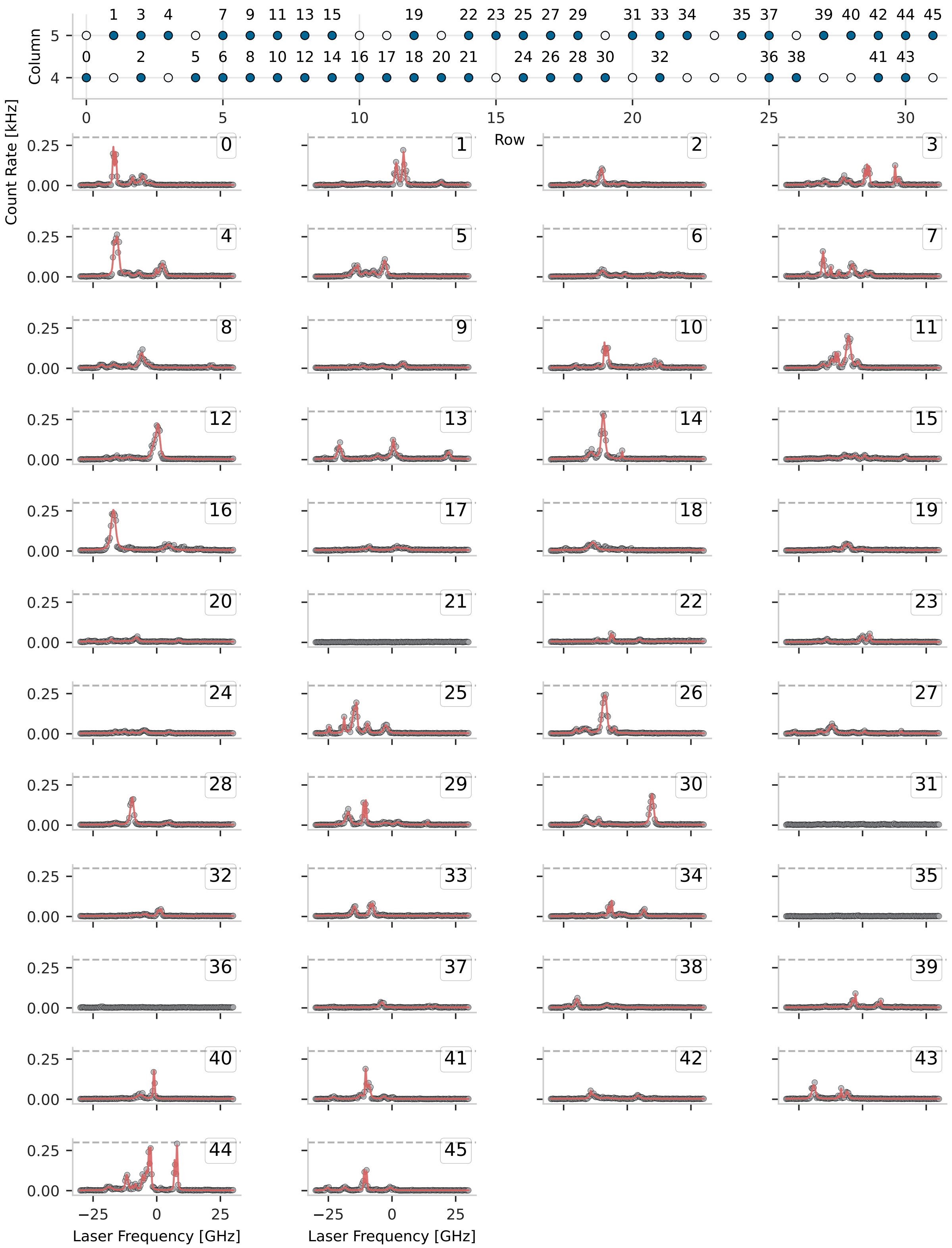

Fig. S10. **Photoluminescence excitation of 64 nanopillars exposed to a single pulse of** $0.18\,\mu J$**.** The white circles indicate a raster of nanopillars. The blue circles indicate pillars with V2 centers that did not pass the threshold, and their respective PLEs are shown below. Note that for all the PLE figures the relative laser frequency was scanned from -30 GHz to 30 GHz (offset is $327.112\,THz$)

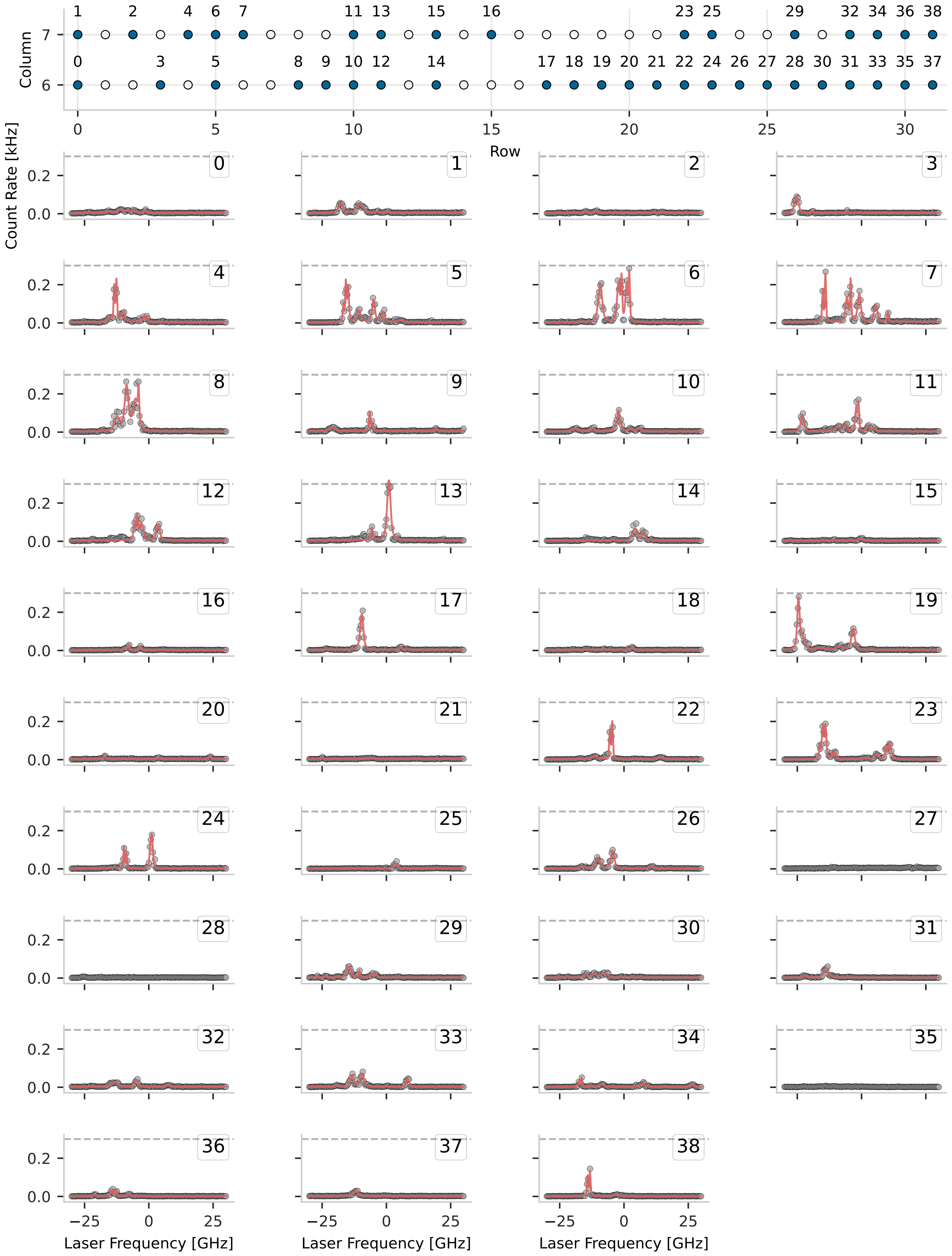

Fig. S11. **Photoluminescence excitation of 64 nanopillars exposed to a single pulse of** 0.24 µJ**.** The white circles indicate a raster of nanopillars. The blue circles indicate pillars with V2 centers that did not pass the threshold, and their respective PLEs are shown below. Note that for all the PLE figures the relative laser frequency was scanned from -30 GHz to 30 GHz (offset is 327.112 THz)

S9. PLE and resonant Hanbury Brown Twiss experiments

For the estimated concentration of V2 centers (PLE peaks) and the measured broad inhomogeneous distribution, we expect that the PLE peaks predominantly correspond to spectrally isolated V2 centers, as is also consistent with the discrete spectral jumps observed (see Fig. S12). To further verify this, we perform additional Hanbury Brown Twiss (HBT) measurements on a set of PLE peaks exceeding a count-rate threshold of 0.3 kHz. During these measurements, we apply low off-resonant excitation power (4 µW) to minimize ionisation and reduce background counts, together with two resonant lasers tuned to the A1 and A2 transitions (each at 50 nW). The HBT measurements show antibunching ($g^{(2)}(0) < 0.5$) for all PLE peaks considered, consistent with the emission predominantly being from a single V2 center, even for PLE peaks with inhomogeneous linewidths of $\sim$2-3 GHz.

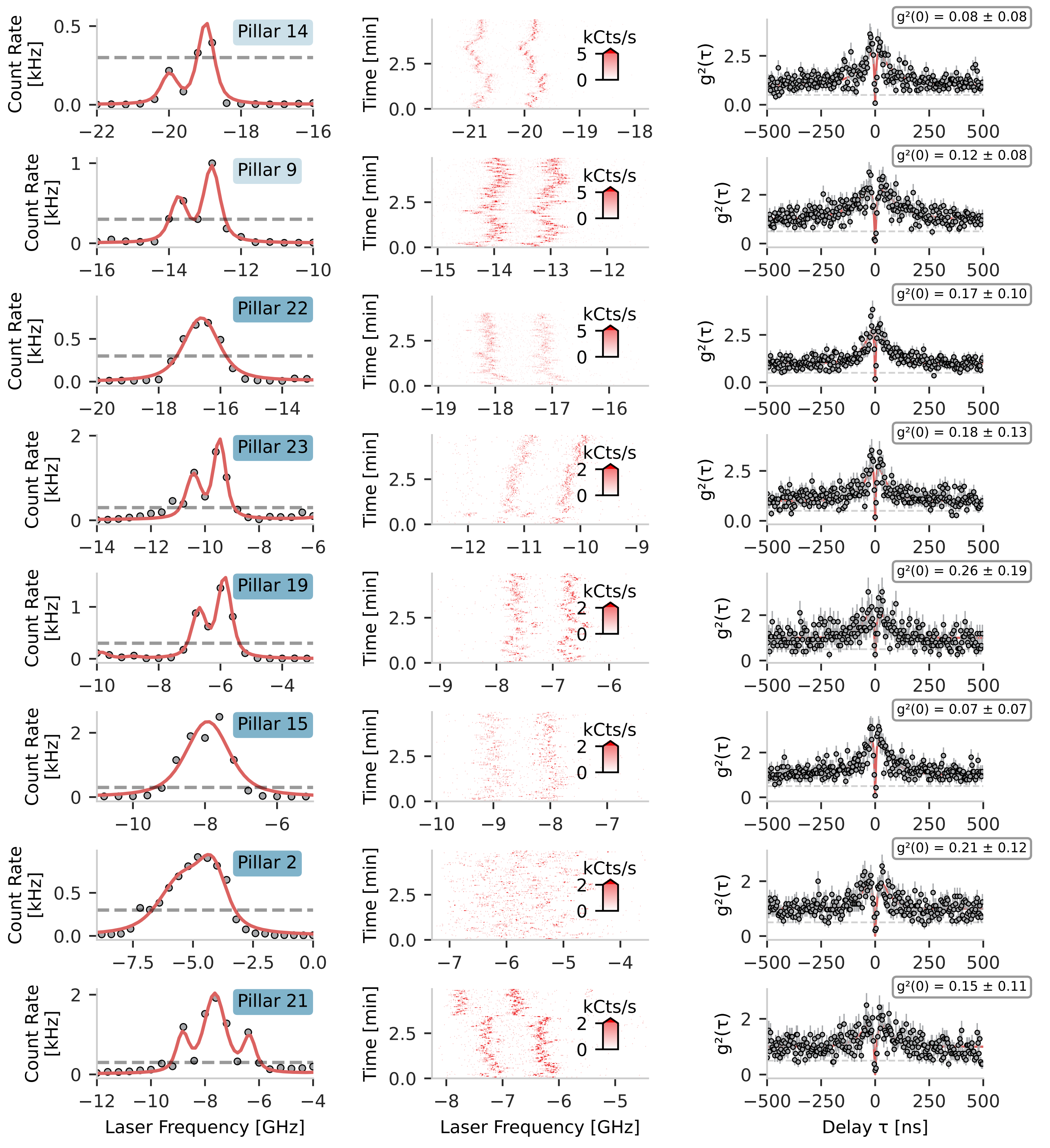

Fig. S12. **PLE and HBT measurements of** V2 **centers. a)** PLE measurements (same scheme as Fig. 2a) for 8 pillars. Light blue corresponds to laser-induced V2 centers in pillars exposed to a single 0.18 µJ UV pulse (see Fig. S7), while dark blue corresponds to pillars exposed to a single 0.24 µJ UV pulse (see Fig. S8). **b)** Scanning-laser PLE measurements (same scheme as Fig. 3c), showing discrete spectral jumps. **c)** HBT measurements under resonant excitation, demonstrating (predominantly) single-photon emission for each PLE peak. As a conservative value we report $g^{(2)}(0)$ that corresponds to the minimum measured data point around $\tau = 0$.

S10. Spectral diffusion rate

To measure the spectral diffusion rate, we employ the scheme shown in S10. Here, we keep both resonant lasers on during the *X-block*, and we use the no-recapture model to fit the data[8]. For off-resonant diffusion, we apply the off-resonant laser during the *X-block* and fit the data to the diffusion-only model[8]. The no-recapture model fits the

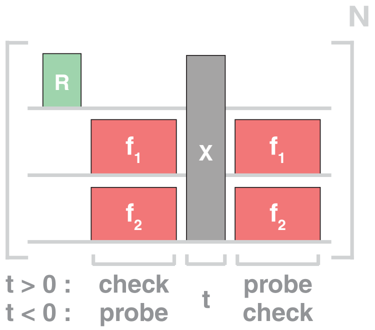

Fig. S13. **Spectral diffusion measurement scheme.** Experimental sequence. A 'check' block (2 ms, 20 nW) is followed by a system perturbation (marked 'X'), which here consists either of turning on the NIR lasers or turning on the off-resonant laser. A second block (2 ms, 20 nW) probes whether the defect has diffused away, or has ionised (denoted 'probe'). Data is post-selected by imposing a minimum-counts threshold (T), heralding the emitter on resonance in the first (second) block and computing the mean number of counts in the second (first) block, which encodes the emitter brightness at future (past) delay times t. To measure spectral diffusion under resonant excitation, we use two resonant lasers, each at 20 nW. For spectral diffusion under off-resonant laser light, we use an off-resonant laser (785 nm with 10 µW

).

data as:

$$C(t)/C_0 = \begin{cases} (1+\gamma_\mathrm{d}\, t\,/\,\Gamma)^{-1}\; e^{-\gamma_\mathrm{i} t}, & \text{if } t>0. \\ (1-\gamma_\mathrm{d}\, t\,/\,\Gamma)^{-1}, & \text{otherwise.} \end{cases} \tag{S4}$$

And the diffusion-only model fits the data as:

$$C(t)/C_0 = \begin{cases} (1+\gamma_\mathrm{d}\, t\,/\,\Gamma)^{-1}, & \text{if } t>0. \\ (1-\gamma_\mathrm{d}\, t\,/\,\Gamma)^{-1}, & \text{otherwise.} \end{cases} \tag{S5}$$

For both models, the spectral diffusion rate γ_d depends on the linewidth of the specific V2 center of interest. We assume that the linewidth is not lifetime-limited, and thus, if we want to extract the diffusion rate, we need to measure the homogeneous linewidth of each V2. Since we did not measure the homogenous linewidth for each V2 center, we take a range for γ_d where:

$$\gamma_\mathrm{d} = \gamma_\mathrm{d,lifetime}\frac{\Gamma}{\Gamma_\mathrm{lifetime}}. \tag{S6}$$

Here, Γ is the homogeneous linewidth of the emitter while $\gamma_\mathrm{d,lifetime}$ is the spectral diffusion rate assuming a lifetime-limited linewidth: Γ_lifetime. We take a maximum diffusion rate for $\Gamma = 3\cdot\Gamma_\mathrm{lifetime}$ (see S11).

S11. Bayesian analysis for Check-Probe PLE

We perform the check-probe PLE around the $A1$ transition to determine the homogeneous linewidths of several V2 centers in the nanopillars of Fig. 3a,b. The pulse sequence for the check-probe ple is shown in Fig. S14. This type of ple allows to measure the homogeneous linewidths in systems where the rate of spectral diffusion is relatively high. Since the check and probe blocks are short (2 ms) compared to the rate of spectral diffusion under resonant excitation.

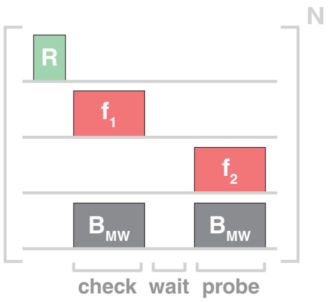

Fig. S14. **Check-probe ple scheme.** An off-resonant laser pulse (10 µs and10 µW) is applied followed by two resonant laser pulses (both 2 ms and 20 nW) together with microwaves (127 MHz) to avoid spinpumping. The first resonant block acts as a check (effective initialisation) and the second resonant block acts as a probe (readout). The two blocks are separated by a waiting time of 5 µs.

The probe spectrum obtained from this sequence typically contains some residual inhomogeneous broadening due to imperfect initialisation in the check step. To account for this, we analyse the data using the Bayesian framework introduced in van de Stolpe *et al.*[8] (Supplementary Note 4). Within this model the mean number counts from the probe block at a frequency detuning **f** is expressed as the convolution of the spectral probability density (i.e. the residual inhomogeneous broadening) and the intrinsic emitter spectral response $\lambda(f)$:

$$C(f) = P(f \mid m \geq T) * \lambda(f)\,, \tag{S7}$$

where the spectral probability density function is given by

$$P(f \mid m \geq T) = \frac{1}{N_T}\left(1 - \Gamma_{\mathrm{i}}\left[T, \lambda(f - f_1)\right]\right)\,, \tag{S8}$$

with $\lambda(f)$ the expected mean number of counts during obtained in a single check block when the emitter is at frequency f and the laser at frequency f_1. The function Γ_{i} denotes the incomplete Gamma function

$$\Gamma_{\mathrm{i}}[a, z] = \frac{1}{\Gamma_{\mathrm{c}}[a]} \int_z^{\infty} t^{a-1} e^{-t} dt \tag{S9}$$

with $\Gamma_{\mathrm{c}}[a]$ the Euler-Gamma function. The emitters initrinsic spectral response is a Lorentzian described as

$$\lambda_{\mathrm{L}}(f) = C_0 \frac{\left(\frac{\Gamma}{2}\right)^2}{f^2 + \left(\frac{\Gamma}{2}\right)^2}. \tag{S10}$$

Because both terms in Equation S7 depend on $\lambda(f)$, the apparent spectral response varies with the set threshold T from the check-block. And thus by increasing T the amount of residual broadening becomes negligible. By fitting Equation S7 to the data where we sweep the threshold, we can extract the homogeneous linewidth Γ and C_0. An example of the fit is shown in Fig. S15.

Threshold sweeps were performed for for several natural V2 centers (Fig. S16) and laser-induced V2 centers (single 0.18 µJ pulse in Fig. S17 and single 0.24 µJ pulse in Fig. S18). The resulting linewidths are compared to the lifetime-limited value of ~26 MHz for the $A1$ transition. Note that due to the initialisation in the check-block with a single laser and a microwaves we also have a small probability to initialise in the $\pm\frac{3}{2}$ subspace. This introduces a small probability of measuring the A2 transition which has a longer lifetime and can therefore result in an lower measured Γ.

For measured two V2 natural V2 centers (different ones from S8 and observe linewidths ~$2 \cdot \Gamma_{\text{lifetime}}$. This indicates that we still have some residual inhomogeneous broadening and/or power broadening effects. Since the measurements were highly automatised, the count rate was not optimised perfectly before each measurement and can influence the spectral density function (thresholding) significantly. Laser-induced V2 centers exhibit comparable $\frac{\Gamma}{\Gamma_{\text{lifetime}}}$ and thus show that the linewidth is not significantly broadened for laser-induced V2 centers and close to the lifetime limit. A more precise determination of the homogeneous linewidth, including Rabi-induced broadening, could be obtained with a low magnetic field (~0 G) and high microwave power, so that Landau-Zener-Stückelberg interference becomes observable. These experiments lie beyond the scope of this study.

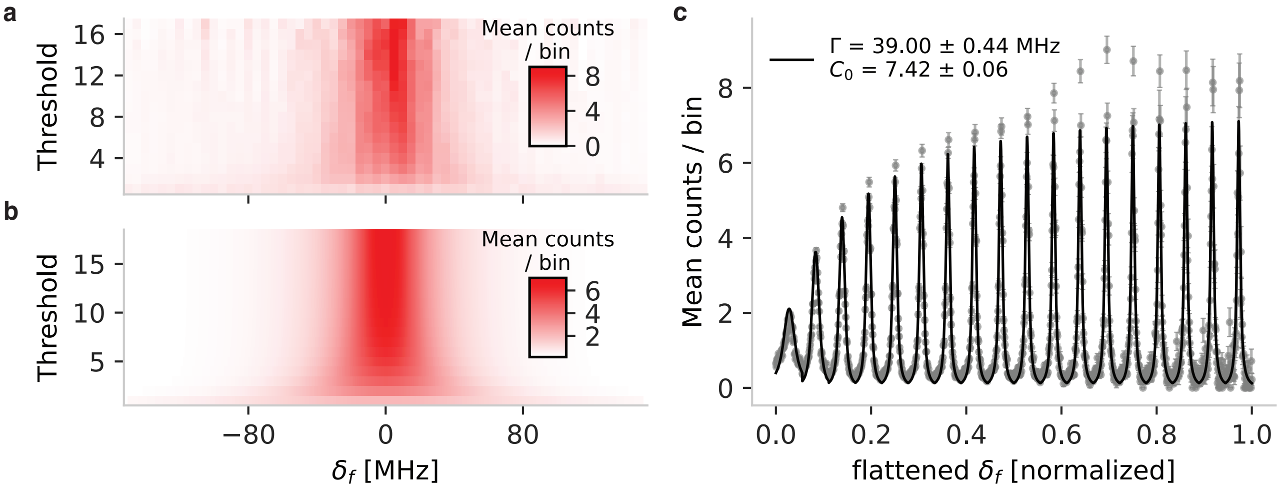

Fig. S15. **Example of fitting check-probe signal. a)** Data obtained from pillar 21 (Fig. S8) where the threshold is swept from 1 to 17. **b)** Fitted convolution of Equation S7 to the data. **c)** Flattened data of a) and b) where the x axis is flattened and normalised. From the fit we extract $\Gamma = 39.0(4)$ MHz and $C_0 = 7.42(6)$

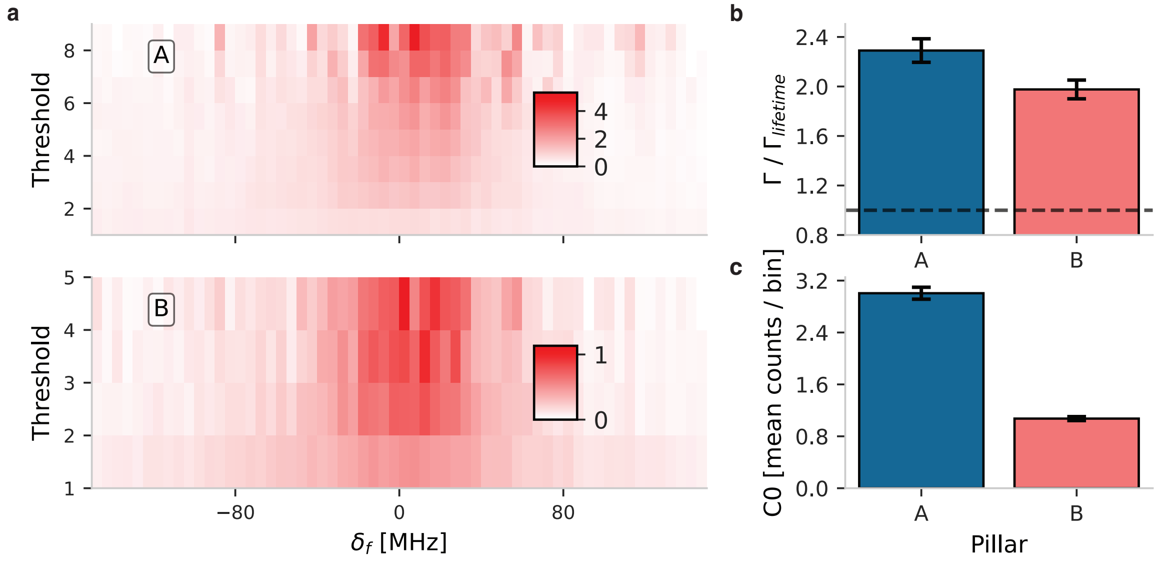

Fig. S16. Γ **for 2 natural** V2 **centers. a)** Data of check-probe ple where threshold is swept for two V2 centers. Which were not in Fig. S6 but a different place. **b)** Extracted Γ from fitting a) compared to Γ_{lifetime} for the same pillars. The dashed line indicates the lifetime limited Γ. **c)** Extracted C_0 from fitting a).

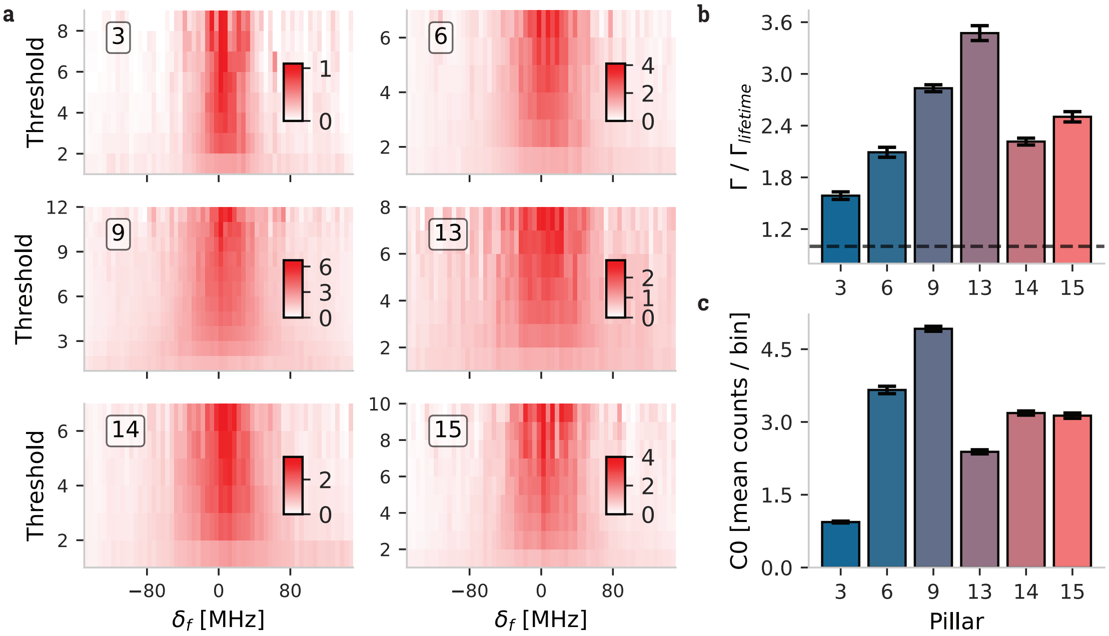

Fig. S17. Γ **for several laser-induced** V2 **centers using UV pulse energy of** 0.18 µJ. **a)** Data of check-probe ple where threshold is swept for six V2 centers. **b)** Extracted Γ from fitting a) compared to Γ_{lifetime} for the same pillars. The dashed line indicates the lifetime limited Γ. **c)** Extracted C_0 from fitting a).

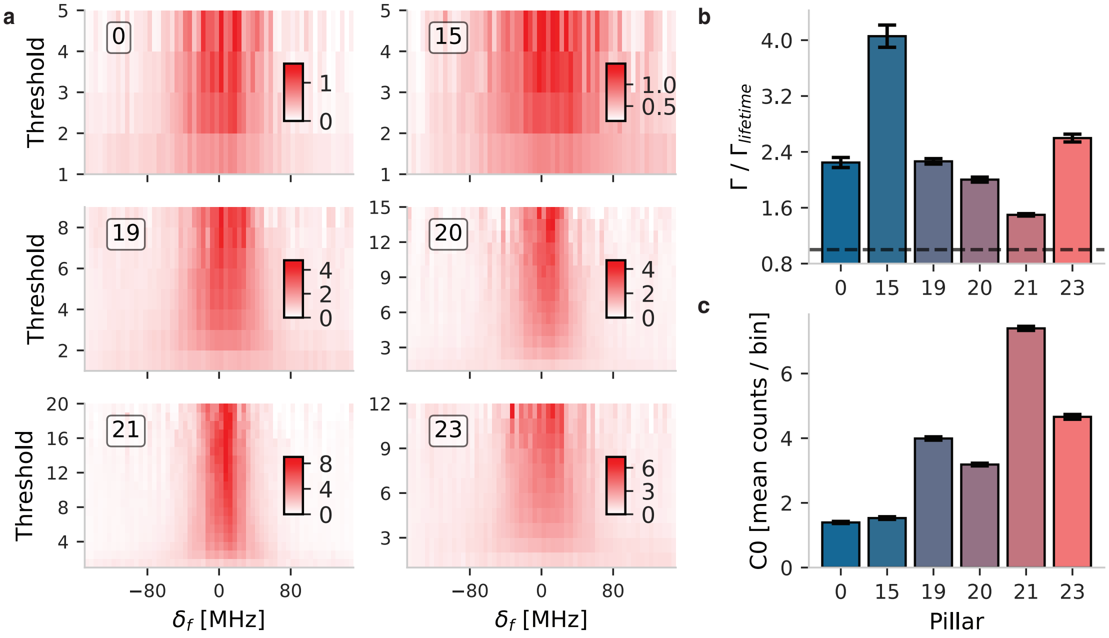

Fig. S18. Γ **for several laser-induced** V2 **centers using UV pulse energy of** 0.24 µJ. **a)** Data of check-probe ple where threshold is swept for six V2 centers. **b)** Extracted Γ from fitting a) compared to Γ_{lifetime} for the same pillars. The dashed line indicates the lifetime limited Γ. **c)** Extracted C_0 from fitting a).

S12. Magnetic field

For the check-probe PLE in Fig. 3d and spin coherence measurements Fig. 4a,b,c, we apply a low magnetic field along the defect symmetry axis (c-axis) using a permanent neodymium magnet outside the cryostat. For the check-probe PLE, we used a magnetic field of approximately ~20 G, and for the spin coherence measurements, we applied a magnetic field of approximately ~40 G so that the $\left|m_s = +\frac{1}{2}\right\rangle \leftrightarrow \left|m_s = +\frac{3}{2}\right\rangle$ transition is around 181.8 MHz. The magnetic field was aligned by applying the sequence depicted in the inset of Fig. 2a without applying MW pulses. We set f_1 to be resonant with the A2 transition and monitor the average photoluminescence (f_1 pulse duration is 2 ms). A (slightly) misaligned field causes spin-mixing between the $m_s = \pm\frac{3}{2}$ and $m_s = \pm\frac{1}{2}$ subspace, which increases the detected signal. Minimising the photoluminescence thus optimises the field alignment along the symmetry axis.

For the T_2^{DD} in Fig. 4d we apply a magnetic field of ~1300 G using a permanent neodymium magnet just behind the sample. We place 3 external magnets outside the cryostat, mounted on motorized stages, to align the magnetic field as described previously.

S13. Optical properties of other laser-induced V2 center

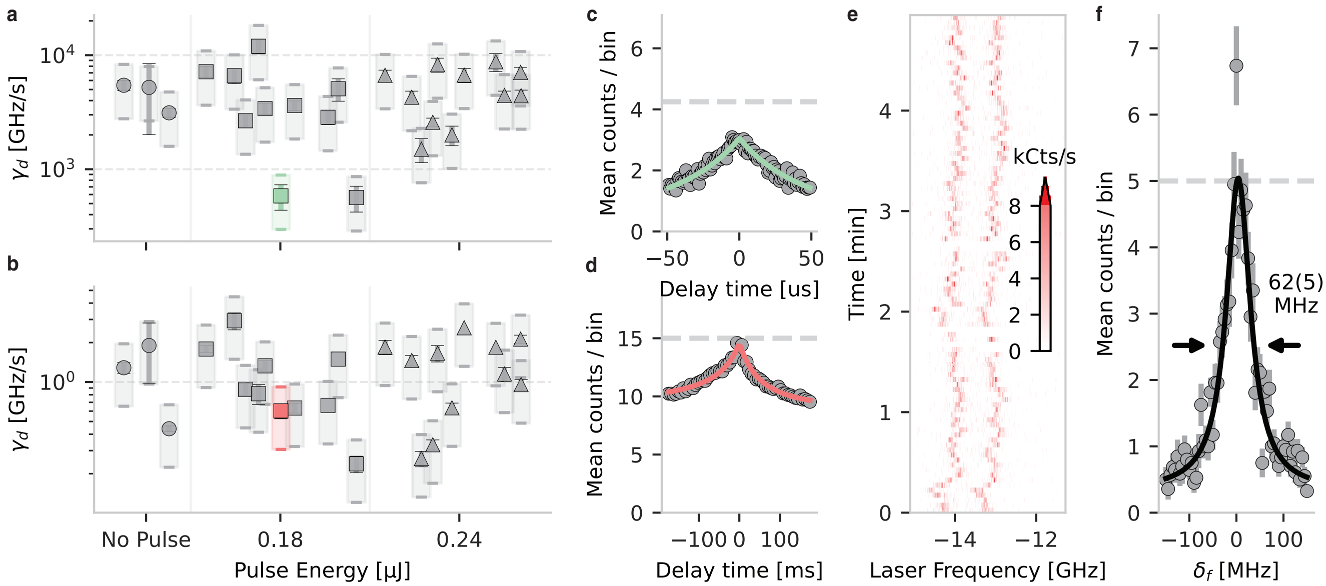

Fig. S19. **Emitter optical properties and laser-induced charge dynamics of** V2 **used for spin coherence measurements. a)** Fitted spectral diffusion constant under offresonant excitation for natural V2 centers (circles) and laser-induced V2 centers (squares and triangles, 0.18 µJ and 0.24 µJ respectively). The error bars indicate error in fit, the shaded region indicates the γ_{d} interval for $1 \leq \Gamma/\Gamma_{\mathrm{lifetime}} \leq 3$ (see S10). γ_{d} is extracted using methodology described in van de Stolpe *et al.*[8]. γ_{d} of the red square indicates the same V2 center as the green square in b) and data in c), d), e) and f). **b)** Fitted spectral diffusion constants under resonant excitation for the same V2 centers as in a). **c)** Data and fit to extract γ_{diff} under off-resonant excitation. **d)** Data and fit to extract γ_{diff} under resonant excitation. **e)** Scanning laser PLE over 5 minutes. The laser is on for a total of 334 ms per scan. We conditionally apply an off-resonant pulse if a single scan gives less than 5 counts. This is done to counter ionisation (several scans show no counts or only a single optical transition). **f** Check-Probe PLE (see van de Stolpe *et al.*[8] for methodology) indicating a near-lifetime limited optical linewidth, performed at a magnetic field of ~20 G

For the measurements on the spin properties, we use one of the brightest V2 centers (pillar 9 Fig. S7). This V2 center also has a relatively low spectral diffusion rate under off-resonant excitation. In Fig. S19 we show the diffusion rates under off-resonant and resonant excitation, together with a scanning PLE and check-probe PLE. We observe a slightly larger linewidth (~$2 \cdot \Gamma_{\mathrm{lifetime}}$ see Supporting Note S11) and slightly faster spectral diffusion rate compared to the V2 center in the main text.

To confirm we are solely addressing a single V2 center we perform a g^2-correlation measurement. We apply limited off-resonant power (1 µW to avoid ionisation and reduce background counts) and two resonant lasers at the frequency of the A1 and A2 transition (both 20 nW and collect data for 20 min)

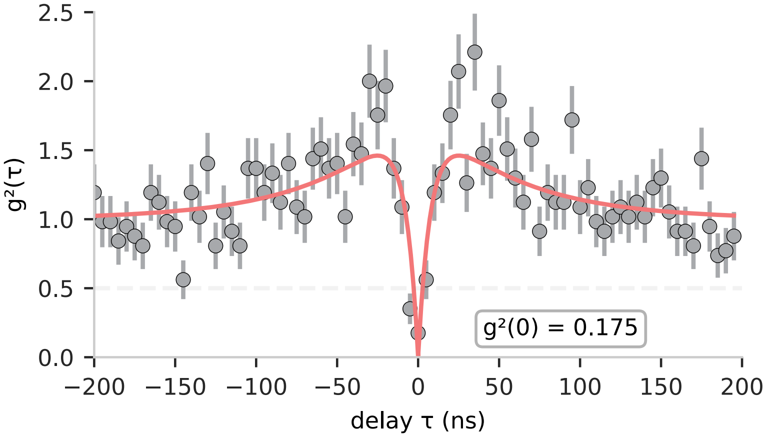

Fig. S20. g^2**-correlation measurement under resonant excitation.** We apply limited off-resonant power (1 µW to avoid ionisation and reduce background counts) and two resonant lasers at the frequency of the A1 and A2 transition (both 20 nW and collect data for 20 min). The data for $g^2(0) = 0.175$ indicates that this is indeed a single V2 center that we can address resonantly.

S14. Electron spin measurements

To investigate the electron spin properties of laser-induced V2 centers, we perform Rabi oscillation, electron spin resonance (ESR), Ramsey, Hahn echo and dynamical decoupling (DD) measurements on pillars 9 and 14(Fig. S7). The pulse sequences for all experiments are summarized in Fig. S21. We begin by resetting the spin environment of the V2 center with a 10 µs 785 nm laser pulse in the **repump** block. Then, we verify that the V2 center is on resonance by illuminating both resonant lasers for 150 µs in the **check** block. Subsequently, we populate the $m_s = \pm\frac{3}{2}$ subspace by exciting the A1 transition for 60 µs in the spin-pump (**sp**) block. This is followed by two identical normalization blocks (**norm.1** and **norm.0**), each exciting the A2 transition for 60 µs. Photon counts are recorded in both blocks. We then apply appropriate microwave pulse sequences to run different measurements in the $\mathbf{MW_M}$ block, before reading out the population in $m_s = \pm\frac{3}{2}$ subspace with a 60 µs A2 laser pulse in the readout (**ro**) block. A wait time of 10 µs is inserted between adjacent blocks.

We apply a threshold on the photon count rates recorded during the **check** block. For those repetitions that pass the threshold, we calculate the mean of photon counts during **norm.1**, **norm.0** and **ro** blocks, denoted as A, B and C respectively, as well as the unbiased estimators of standard deviations σ_A, σ_B and σ_C. The normalized readout R is then calculated as

$$R = \frac{C - B}{A - B}, \tag{S11}$$

and the uncertainty σ_R is propagated as

$$\sigma_R = \sqrt{\frac{(C-B)^2}{(A-B)^4}\sigma_A^2 + \frac{(A-C)^2}{(A-B)^4}\sigma_B^2 + \frac{1}{(A-B)^2}\sigma_C^2}. \tag{S12}$$

In ESR measurements, we fit the normalized readout R to 2 Gaussian peaks with a shared full-width at half-maximum (FWHM). We then extract a T_2^* using

$$T^*_{2,\mathrm{DESR}} = \frac{2\sqrt{\ln 2}}{\pi \cdot \mathrm{FWHM}}. \tag{S13}$$

In detuned Ramsey measurements, we fit the normalized readout R to the following function:

$$R = b + \mathrm{e}^{-(\tau/T_2^*)^2} \sum_i A_i \cos\left[\left(f_c + \frac{(-1)^i}{2} f_{\mathrm{HF}}\right)\tau + \varphi_i\right], \tag{S14}$$

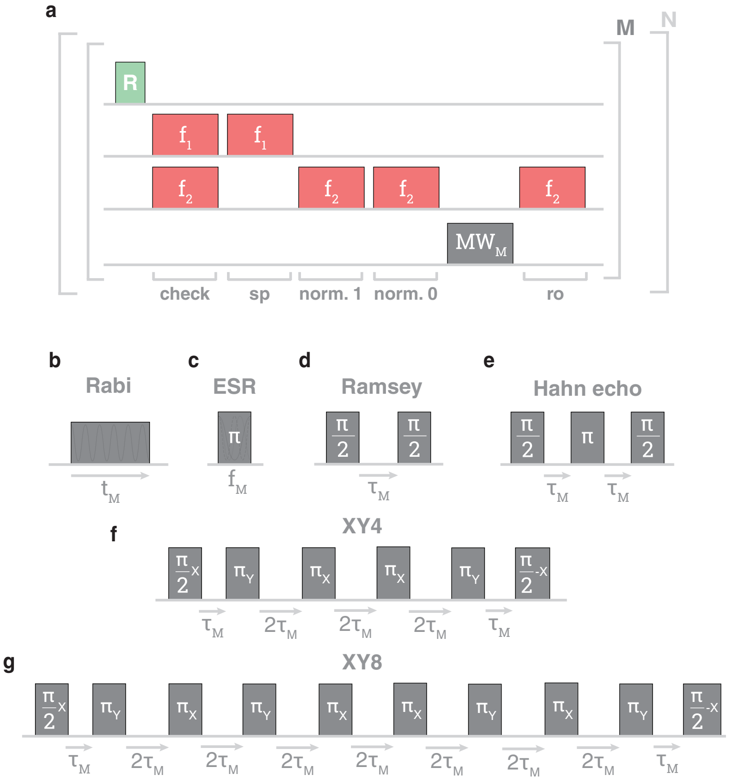

Fig. S21. **Pulse schemes for spin measurements. a)** A framework of pulse sequences shared by Rabi, electron spin resonance (ESR), Ramsey, Hahn echo, and dynamical decoupling (DD) measurements is reported in the main text. R represents a 785 nm repump laser, f_1 a laser resonant with the A1 transition, f_2 a laser resonant with the A2 transition, and MW_M some parametrized microwave sequence that depends on the specific measurement and the parameter M. In each measurement, we run the entire laser and microwave sequence M times, sweeping the parameter M for the MW sequence, and we repeat the sweep N times to mitigate errors such as shot noise. **b)** In Rabi measurements, we fix the frequency and power of MW driving and sweep its duration t_M. **c)** In ESR, we send a MW π-pulse for fixed duration and power and sweep its frequency f_M. **d)** In Ramsey measurements, we sweep MW sequences of the form $(\frac{\pi}{2} - \tau_M - \frac{\pi}{2})$, where $\pi/2$ represents a MW $\pi/2$-pulse with fixed duration, frequency and power and τ_M represents the variable interpulse delay. **e)** In Hahn echo measurements, we sweep MW sequences of the form $(\frac{\pi}{2} - \tau_M - \pi - \tau_M - \frac{\pi}{2})$, with $\frac{\pi}{2}$, π and τ_M similarly defined. **f) g)** In DD measurements, we run XY4 and XY8 sequences and sweep the interpulse delay $2\tau_M$. $\frac{\pi}{2}_X$ and $\frac{\pi}{2}_{-X}$ represent MW $\frac{\pi}{2}$ pulses with a phase of 0° and 180° respectively, and π_X and π_Y represent MW π pulses with a phase of 0° and 90° respectively,

where τ is the interpulse delay, $i = 0, 1$ labels the peaks fitted in the DESR spectrum, A_i and φ_i are the amplitude and phase of the oscillation due to detuning from each DESR peak, f_c is the center or average frequency of all oscillations, f_{HF} is the hyperfine coupling of the nuclear spin, b is a global offset and T_2^* is the extracted dephasing time. For the fit in the main text (Fig. 4c) we extract $f_c = 2.13(3)$ MHz.

In Hahn echo measurements, we fit the normalized readout R to a stretched decay function:

$$R = b + A\mathrm{e}^{-(t/T_2)^n}, \tag{S15}$$

where $t = 2\tau$ is the total time the V2 electron spin is allowed to dephase on the xy-plane of the Bloch sphere, τ is the interpulse delay, A is the amplitude of the decay, b is a global offset, T_2 is the extracted coherence time and n is the decay exponent.

In DD experiments, we fit the same stretched decay function. Here, we have $t = 2\mathrm{N}\tau$ where N is the number of decoupling π pulses and 2τ is the wait time between two successive π pulses.

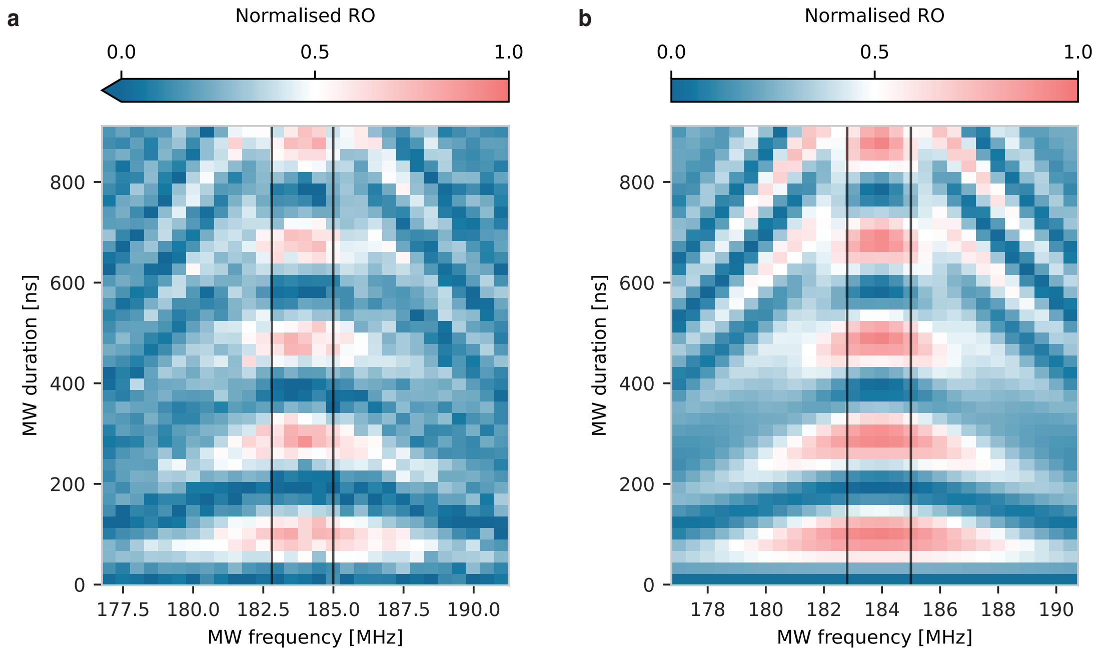

Fig. S22. **Measured and simulated Rabi Chevron patterns of Pillar 9.** Solid dark lines in each panel correspond to resonant frequencies fitted in the DESR spectrum (Fig. 4b in main text). **a)** Measured data, also displayed in Fig. 4a. **b)** Simulated with the hyperfine coupling extracted from the DESR spectrum.

S15. Spin coherence of V2 center in pillar 14 at low magnetic field

We also characterised the spin properties of the highlighted V2 center in Fig. 3 (pillar 14 Fig. S7). For this V2 center, we could not find a (resolvable) strongly coupled nuclear spin. See Fig. S23a,b for the Rabi chevron pattern and ESR measurement. We find a similar spin coherence time $T_2^* = 1.3(2)$ µs (see Fig. S23c) as the V2 center shown in Fig. 4.

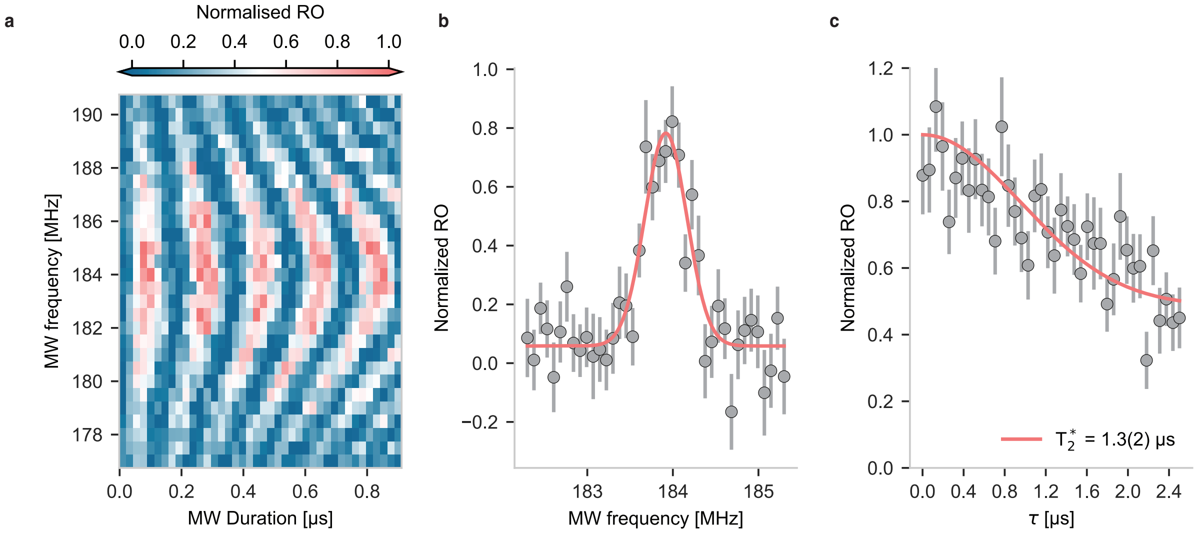

Fig. S23. **Spin measurements at low field of** V2 **center in pillar 14: a)** Rabi chevron pattern. Details on the measurement sequence and normalisation procedure are elaborated in S14. **b)** Electron-spin-resonance of V2 center revealing no strongly coupled nuclear spin. **c)** Resonant Ramsey measurement on the same V2 center with $f_{MW} = 1814.8$ MHz. Red line indicates a fit to a Gaussian decay with $T_2^* = 1.3(2)$ µs.

S16. Optical setups

For this work, we used 2 confocal setups, one at both ambient pressure and temperature, and one cryogenic setup (4K). All data was taken using the QMI Python package.[9]

a. Ambient-temperature setup

We couple an 785 nm off-resonant laser (Cobolt 06-MLD785) to free space using a zoom fiber collimator (Thorlabs, ZC618APC). Free Space: The collimated beam passes through a variable neutral density filter (ND, Thorlabs NDC-50C-4-B), after which a $\lambda/2$—$\lambda/4$ waveplate combination allows for polarisation control. The excitation path and detection path are separated with a 925 nm dichroic beamsplitter (Semrock FF925-Di01-25x36). We use a nanosecond-pulsed nitrogen laser (LaserTechnik Berlin MNL 100) which delivers 3 ns pulses at 337.1 nm. We control the energy of the UV pulses using a neutral density filter on a servo motor (coarse) and the laser's voltage (fine). We measure the pulse energy just before the objective using a μ-Joule Meter (PEM 250, LaserTechnik Berlin). Using a 355 dichroic beamsplitter (Semrock, Di01-R355-25x36) we align the UV laser to the sample. A flip mirror and 50/50 pellicle beamsplitter (Thorlabs, BP150) enable imaging of the sample with a visible LED (MCWHL6-C2) and a CCD-camera (ClearView Imaging, BFS-U3-16S2M-CS). Two different objectives are used to focus the laser onto the sample: a 60X, 0.7 NA (Olympus LUCPLFLN60X) and a 100×, 0.9 NA objective ([137]). The 60X, 0.7 NA objective is employed for laser writing because of its compatibility with UV wavelengths. For room-temperature defect characterization, we use the 100X, 0.9 NA objective, which offers improved photon collection, but does not support UV transmission. The objective can be moved using a configuration of 3 piezo-electric stages (PI Q545.140). Collected fluorescence passes through both dichroic beamsplitters and is filtered with an 830 nm long-pass filter (Semrock, BLP01-830R-25) and collected in a fiber. We perform the 2D-PL measurements without a 50/50 beamsplitter, but for the saturation measurements, we used a 50/50 beamsplitter as we also performed g^2-correlation measurements. We use two single-photon superconducting nanowire single-photon detectors (SNSPDs, Single Quantum) with over 90% detection efficiency at 917 nm. The coarse time scheduling (1 s resolution) of the experiments is managed by a microcontroller (ADwin Pro II)

b. Low-temperature optical setup

The low-temperature optical setup is divided into two parts, in-fiber (left) and free-space (right). The electronics are not depicted in the figure.

In Fiber: Two NIR lasers (916 nm, Toptica DL Pro and the Spectra-Physics Velocity TLB-6718-P, are frequency-locked to a wavemeter (HF-Angstrom WS/U-10U), using a 99/1 beamsplitter. Their optical power is modulated by acousto-optic modulators (AOM, G&H SF05958). The power of the 785 nm repump laser (Cobolt 06-MLD785) is directly controlled via analog modulation. A wavelength division multiplexer (WDM, OZ Optics) combines the 785 nm repump and 916 nm NIR laser light, after which the light is coupled out to free space using a zoom fiber collimator (Thorlabs, ZC618APC).

Free Space: The collimated beam passes through a variable neutral density filter (ND, Thorlabs NDC-50C-4-B), after which a shortpass filter at an angle (Semrock, FF01-945/SP-25) is used to remove any residual noise from the NIR lasers. A $\frac{\lambda}{2} - \frac{\lambda}{4}$ waveplate combination allows for polarisation control. The excitation and detection paths are separated by a broadband 90:10 beamsplitter (Thorlabs, BS041). We use a fast-steering mirror (Newport, FSM-300-02) and a 4f system to scan the lasers over the sample. A flip mirror and 50/50 pellicle beamsplitter (Thorlabs, BP150) enable imaging of the sample with a visible LED (MCWHL6-C2) and a CCD-camera (ClearView Imaging, BFS-U3-16S2M-CS).

A 0.9 NA microscope objective (Olympus, MPLFLN 100X) is used to focus excitation light onto the nanopillars and to collect fluorescence. The objective is kept at room temperature and under vacuum, and is moved (coarse) using a configuration of 3 piezo-electric stages (PI Q545.140). The sample is cooled down to 4 K in a closed-cycle cryostat (Montana Instruments S100), while a heat shield kept at 30 K limits thermal radiation from the objective.

Collected fluorescence passes through a 90/10 beamsplitter, after which it can be routed either to a spectrometer (Princeton Instruments IsoPlane 81), filtered with an 830 nm long-pass filter (Semrock, BLP01-830R-25), or to the single photon superconducting nanowire single-photon detectors (SNSPDs, Single Quantum) with over 90% detection efficiency at 917 nm. Next to a 830 nm long pass filter, an additional long-pass filter (Semrock, FF01-937/LP-25), placed at an angle, is used to filter out reflected light originating from the NIR lasers (916 nm).

Electronics: Microwave pulses are generated with an arbitrary waveform generator (Zurich Instruments, HDAWG8) and subsequently amplified (Mini-circuits LZY-22+). A bondwire is spanned across the sample to deliver the MW

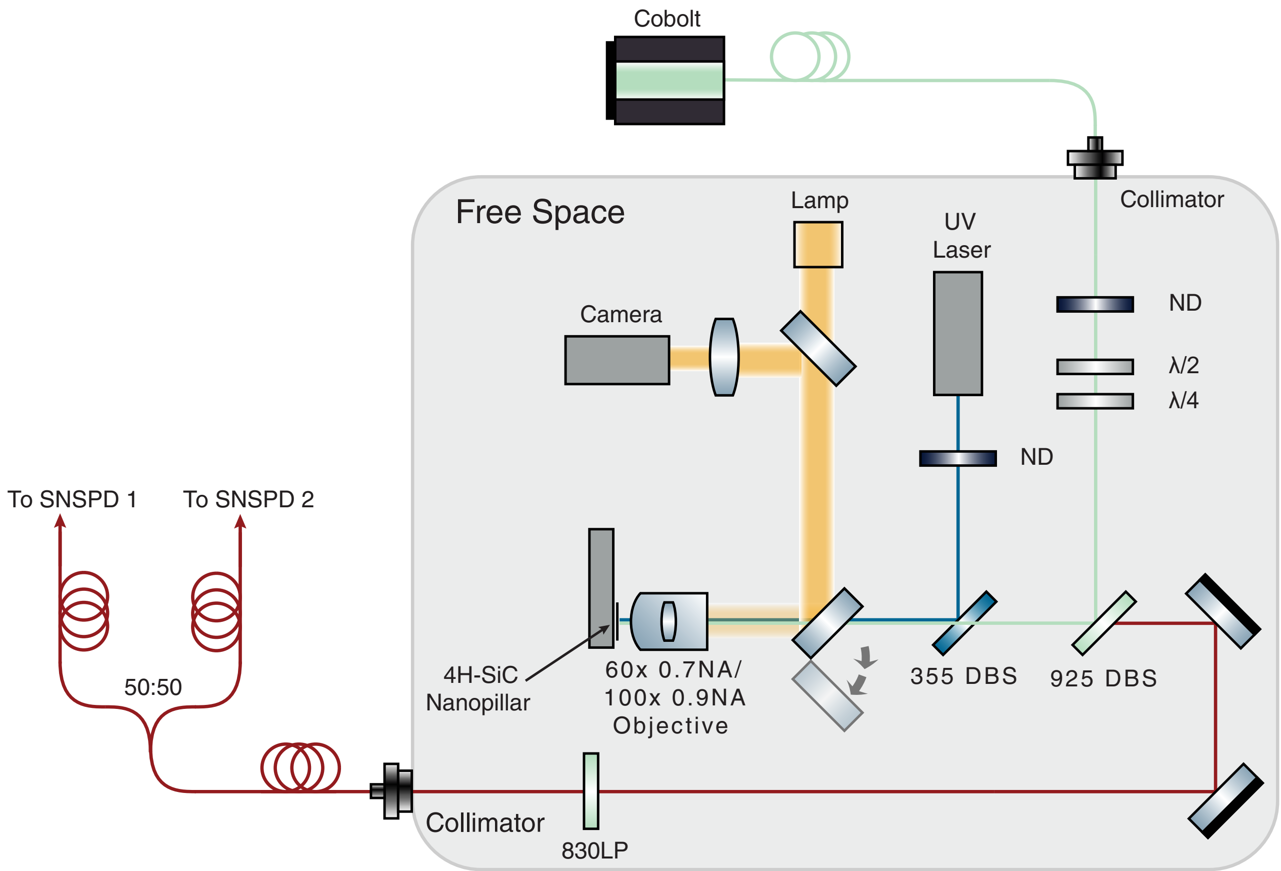

Fig. S24. **Room-temperature confocal optical setup.** Details in text above the figure.

radiation close to the sample surface (~25 µm). The coarse-time scheduling (1 µs resolution) of the experiments is handled by a microcontroller (ADwin Pro II).

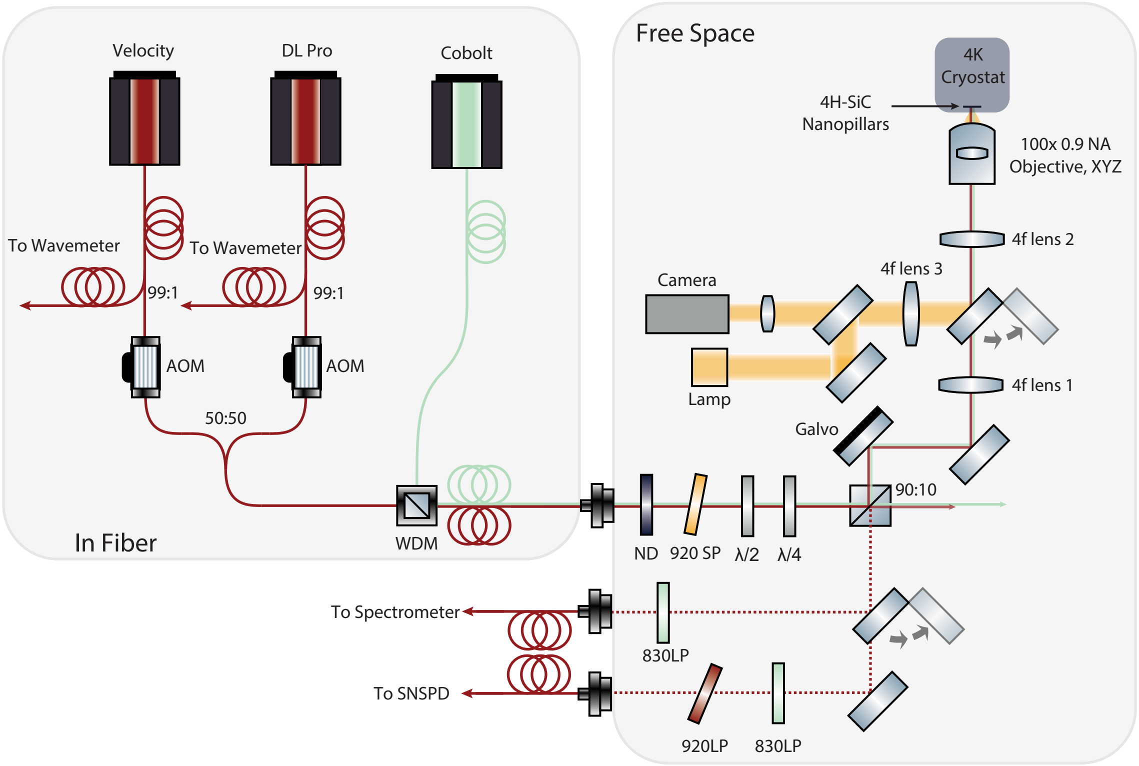

Fig. S25. **Low-temperature confocal optical setup.** Details in text above the figure.

[1] Son, N. T.; Ivanov, I. G. Charge State Control of the Silicon Vacancy and Divacancy in Silicon Carbide. *Journal of Applied Physics* **2021**, *129*, 215702.

[2] Nagy, R. et al. High-Fidelity Spin and Optical Control of Single Silicon-Vacancy Centres in Silicon Carbide. *Nature Communications* **2019**, *10*, 1954.

[3] Heiler, J.; Körber, J.; Hesselmeier, E.; Kuna, P.; Stöhr, R.; Fuchs, P.; Ghezellou, M.; Ul-Hassan, J.; Knolle, W.; Becher, C.; Kaiser, F.; Wrachtrup, J. Spectral Stability of V2 Centres in Sub-Micron 4H-SiC Membranes. *npj Quantum Materials* **2024**, *9*, 34.

[4] Wood, R. M. *Laser-Induced Damage of Optical Materials*; CRC Press: Boca Raton, 2003.

[5] Schaffer, C. B.; Brodeur, A.; Mazur, E. Laser-Induced Breakdown and Damage in Bulk Transparent Materials Induced by Tightly Focused Femtosecond Laser Pulses. *Measurement Science and Technology* **2001**, *12*, 1784.

[6] Balling, P. *Handbook of Laser Micro- and Nano-Engineering*; Springer, Cham, 2020; pp 1–58.

[7] Singh, S.; Potopowicz, J. R.; Van Uitert, L. G.; Wemple, S. H. Nonlinear Optical Properties of Hexagonal Silicon Carbide. *Applied Physics Letters* **1971**, *19*, 53–56.

[8] van de Stolpe, G. L.; Feije, L. J.; Loenen, S. J. H.; Das, A.; Timmer, G. M.; de Jong, T. W.; Taminiau, T. H. Check-Probe Spectroscopy of Lifetime-Limited Emitters in Bulk-Grown Silicon Carbide. *npj Quantum Information* **2025**, *11*, 31.

[9] Ervasti, H. et al. QMI - Quantum Measurement Infrastructure, a Python 3 Framework for Controlling Laboratory Equipment. *4TU.ResearchData* **2026**,
